# Confining Flat Ru Islands into TiO_2_ Lattice with the Coexisting Ru–O–Ti and Ru–Ti Bonds for Ultra‐Stable Hydrogen Evolution at Amperometric Current Density and Hydrogen Oxidation at High Potential

**DOI:** 10.1002/advs.202410881

**Published:** 2024-10-25

**Authors:** Luyun Chen, Chunlei Li, Mengling Liu, Ziruo Dai, Haibin Wang, Xuan Zhou, Qiuping Zhao, Yuanyuan Cong

**Affiliations:** ^1^ School of Petrochemical Technology Lanzhou University of Technology Lanzhou Gansu 730050 China; ^2^ Key Laboratory of Low Carbon Energy and Chemical Engineering of Gansu Province Lanzhou University of Technology Lanzhou Gansu 730050 China

**Keywords:** hydrogen energy conversion, interfacial Ru–O–Ti bond, interfacial Ru–Ti bond, lattice confined Ru, synergistic effect

## Abstract

Effective hydrogen evolution reaction (HER) under high current density and enhanced hydrogen oxidation reaction (HOR) over a wide potential range remain challenges for Ru‐based electrocatalysts because its strong affinity to the adsorbed hydroxyl (OH_ad_) inhibits the supply of the adsorbed hydrogen (H_ad_). Herein, the coexisting Ru─O─Ti and Ru─Ti bonds are constructed by taking TiO_2_ crystal confined flat‐Ru clusters (F‐Ru@TiO_2_) to cope with above‐mentioned obstacles. The different electronegativity (χ_Ti_ = 1.54 < χ_Ru_ = 2.20< χ_O_ = 3.44) can endow Ti in Ru─O─Ti bonds with more positive charge and stabilize Ru of Ru–Ti bonds with the low‐valence. The strength of Ru─OH_ad_ is then weakened by the oxophilicity of positively charged Ti in Ru─O─Ti bonds and the stronger Ti─OH_ad_ bond could release active Ru, especially for low‐valence Ru in Ru─Ti bonds, to serve as exclusive H_ad_ sites. As expected, F─TiRu@TiO_2_ shows a low HER overpotential of 74 mV at 1000 mA cm^−2^ and an ultrahigh mass activity (j_0,m_) of 3155 A g_Ru_^−1^ for HOR. More importantly, F─Ru@TiO_2_ can tolerate the HER current density of 1000 mA cm^−2^ for 100 h and the high anodic potential for HOR up to 0.5 V versus RHE.

## Graphical abstract

The developed flat Ru islands embedded into TiO_2_ lattice (F‐Ru@TiO_2_) show ultra‐high hydrogen evolution reaction (HER) and hydrogen oxidation reaction (HOR) performance due to the co‐existing RuOTi and RuTi bonds. The electron‐deficient Ti atoms within the RuOTi bonds preferentially favor the adsorption of hydroxyl species (OH_ad_), freeing up more accessional Ru active centers. Especially in the RuTi bonds, the electron‐rich Ru atoms exhibit unfavorable affinity for OH_ad_, making these sites particularly well‐suited for H adsorption (H_ad_).

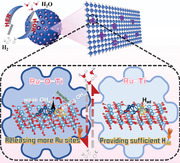

## 1 Introduction

Electrocatalytic water splitting and hydrogen fuel cell are emerging as innovative solutions to address environmental and climate issues.^[^ ^1^, ^2^ ^]^ The former provides a green method for hydrogen production and the latter is a prominent application of hydrogen energy source. To achieve large‐scale hydrogen and perform an efficient hydrogen‐utilization, electrocatalysis for alkaline media have attracted more attention in terms of their lower corrosion than that in acidic media.^[^ ^3^, ^4^ ^]^ Unfortunately, two important reactions, the alkaline hydrogen oxidation reaction (HOR) and hydrogen evolution reaction (HER), present slow kinetic process over the benchmark Pt electrocatalyst.^[^ ^5^, ^6^ ^]^ It is thus highly desired to develop efficient Pt‐free electrocatalysts for alkaline HOR/HER.

Recently, Ru is an appealing alternative to Pt due to enhanced hydroxyl (OH_ad_) binding energy and high water dissociation efficiency, but much lower cost.^[^ ^7^ ^]^ However, the strong coupling effect of orbitals of Ru 3d and O 2p makes Ru too oxyphilic, resulting in the limitation of OH_ad_ transfer in the Volmer reaction, deteriorating its intrinsic activity in the alkaline HOR/HER.^[^ ^8^, ^9^ ^]^ Moreover, the progressive accumulation of Ru‐OH_ad_ species within the inner/outer‐Helmholtz planes would inhibit the adsorption of reactive H_ad_ for the alkaline HOR at elevated anodic potential and hamper re‐formation of H_ad_ intermediates for the alkaline HER at large electrocatalytic current densities.^[^ ^10^, ^11^ ^]^ As a result, most of the Ru‐based electrocatalysts still suffer from low current density at the potential above ∼0.1 V versus (vs.) RHE under the harsh oxidative environment on anodic HOR sides and exhibit high overpotentials at industry‐level large current density for alkaline HER.^[^ ^12^, ^13^, ^14^ ^]^ Therefore, freeing the metallic Ru surface from OH_ad_ and enabling these centers occupied by H_ad_ are essential for the good HOR/HER performance of Ru‐based electrocatalysts.^[^ ^15^, ^16^ ^]^

To this end, constructing the composite by integrating Ru with strongly oxophilic metal oxide to generate well‐defined interface is an effective strategy to compensate for the drawback of single Ru sites. And, the surface electronic features and energy level of Ru can also be modified by interfical chemical bonds, resulting in an unusual reactivity.^[^ ^17^ ^]^ Through building Ru‐O‐Mo bonds at Ru/MoO_2_, the oxyphilic Mo sites can alleviate the over‐adsorption of OH_ad_ on Ru sites by competitive adsorption of OH_ad_ between Ru and MoO_2_, leading to a low overpotential for alkaline HER.^[^ ^18^, ^19^, ^20^ ^]^ Recently, Sun and Feng et al. developed a composite of Ru dopants in the SnO_2_ substrate, in which the interaction between Ru single‐atoms and SnO_2_ by Ru‐O‐Sn bonds favorably transfered the strong adsorption of OH_ad_ on the Ru, promoting the Volmer step of alkaline HER.^[^ ^21^ ^]^ However, Ru/metal‐oxide is usually not a good bifunctional hydrogen electrocatalyst. Thus far, most reported Ru/metal‐oxide electrocatalysts such as MoO_x_‐Ru,^[^ ^22^ ^]^ Ru‐CeO_2_/C^[^ ^23^ ^]^ tend to have interfacial Ru‐O‐M bonds, HOR current density is lower than that of Pt/C after the potential is increased above 0.1 V vs. RHE due to an unbalance between facilitating OH_ad_ formation and preventing the OH_ad_ poisoning. Notably, when a large number of Ru–Ti bonds are formed at the interface by Ru diffusing into TiO_2_ lattice, Ru@TiO_2_ can effectively catalyze the alkaline HOR up to a potential of 0.9 V vs. RHE since electron migration from TiO_2_ to Ru metal causes the release of more active Ru atoms for the adsorption of H (H_ad_).^[^ ^24^ ^]^ In addition, Chen et al. demonstrated that Mn_1_O_x_(OH)_y_@Ru/C with interfacial Ru‐Mn coordination was not easily damaged and passivated by O species during HOR process.^[^ ^25^ ^]^ Till now, the regulation means (such as interfacial Ru‐O‐M engineering and Ru‐M bonds engineering) for electronic structure or band structure of Ru are separate. As a result, the field of Ru‐based bifunctional electrocatalysts with excellent HER activity at large current density and high HOR current in a wide potential range is almost blank. This is likely to combine different interfacial chemical bonds in a Ru/metal‐oxide nanohybrid, inheriting the advantages from each bond. For the Ru‐O‐M bonds, Ru and M such as Ti with low electronegativity relative to O would preferentially serve as Lewis acid sites for selective OH^−^ adsorption due to electrons migrating from the less electronegative Ru/Ti to the more electronegative O via the bridging ligand, resulting in their positively charged surface.^[^ ^26^ ^]^ For the Ru‐M bonds, M metal such as Ti exhibits a relatively low electronegativity compared with Ru, which facilitates electron transfer from Ti to Ru, eventually preventing Ru from binding oxygen‐containing species and providing active low‐valence sites for the formation of Ru‐H_ad_.^[^ ^27^ ^]^ Previous work has also confirmed that Ru‐O_4_ coordination configuration as Lewis acid site closely cooperates with metallic Ru clusters to accelerate hydrogen energy conversion by efficiently providing dual‐sites for OH_ad_ and H_ad_ intermediates, respectively.^[^ ^28^, ^29^ ^]^ Nevertheless, the cooperative construction of interfacial Ru‐O‐M and Ru‐M bonds to adjust the OH adsorption and promote the efficient adsorption‐desorption of H is rarely studied.

Herein, a unique flat Ru islands embedded into the lattice of TiO_2_ electrocatalyst (F‐Ru@TiO_2_) with the co‐existing Ru–O–Ti and Ru–Ti bonds is reported for the first time. The electron‐deficient environment on the Ti atom in Ru–O–Ti bonds results in preferential adsorption of OH_ad_ at the Ti site, which can effectively boosts the OH_ad_ transfer on Ru sites, releasing more accessional Ru active centers for H adsorption. Especially in Ru–Ti bonds, the Ru atoms with the electron‐rich environment causes unfavorable affinity towards OH_ad_ species due to the the repulsive force between its surface electrons and lone pair electrons of O atoms, enabling it to serve as specialized H adsorption sites. As a result, the F‐Ru@TiO_2_ as a HER electrocatalyst can afford an overpotential of only 74 mV at 1000 mA cm^−2^, along with long‐term stability exceeding 100 h. In addition, the electrocatalyst not only achieves excellent HOR j_0,m_ (3155 A g_Ru_^−1^), but also displays catalytic HOR current at as high as 0.5 V vs. RHE. This work sheds light on accurately designing the specific interfacial perimeters for the development of efficient and robust hydrogen energy conversion electrocatalysts.

## 2 Results and Discussion

### 2.1 Materials Synthesis and Physical Characterization

**Figure 1:**
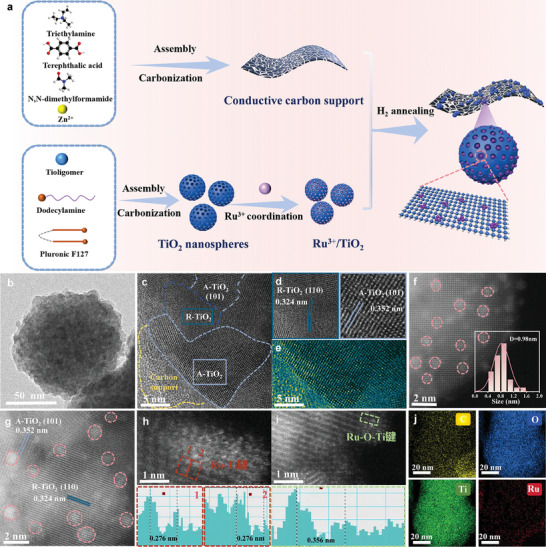
a) Schematic illustration of the synthesis of F‐Ru@TiO_2_. b) TEM, c,d) HRTEM, e) filtered image, f,g) HAADF‐STEM, h,i) aberration‐corrected HAADF‐STEM, and j) EDS elemental mapping images of F‐Ru@TiO_2_.

The flat Ru islands epitaxially grown on TiO_2_ lattice were synthesized via a three‐step, as described in **Figure** **1**a. First, the prepared Zn‐MOF nanosheets were carbonized through high‐temperature pyrolysis to generate conductive carbon support (Figure S1a, Supporting Information). Then, oxygen vacancy‐enriched TiO_2_ nanospheres were obtained by the double‐surfactant‐assisted assembly sol‐gel process, followed by annealing in a N_2_ atmosphere (Figure S1b, Supporting Information).^[^ ^30^ ^]^ Soon afterward, the Ru salt and oxygen vacancy‐enriched TiO_2_ nanospheres were mixed together with conductive carbon support in sequence. TiO_2_ nanospheres with strong coordination capability due to rich oxygen vacancy can well disperse Ru^3+^ metal ions on its defective surface. Finally, low‐temperature reduction in the H_2_/Ar skillfully confined flat Ru islands into the TiO_2_ lattice (Figure S1c,d, Supporting Information). Similarly, electrocatalysts composed of hemispherical Ru islands on TiO_2_ (namely HS‐Ru@TiO_2_, Figure S1e, Supporting Information) by hydrothermal reduction processs and with no TiO_2_ addition (namely Ru/C, Figure S1f, Supporting Information) were also synthesized for comparison. The energy‐dispersive X‐ray spectroscopy (EDS) reveals the loading of Ru in F‐Ru@TiO_2_, HS‐Ru@TiO_2_, and Ru/C as 9.11, 10.38, and 9.78 wt%, respectively (Figure S2, Supporting Information).

The scanning electron microscopy (SEM, Figure S1c, Supporting Information) and transmission electron microscopy (TEM, Figure 1b) image reveal the as‐made F‐Ru@TiO_2_ electrocatalyst is rough with relatively distinct spherical morphology. High‐resolution transmission electron microscope (HRTEM) image shows that there are two kinds of different crystal faces on a region (Figure 1c). The lattice fringes of 0.352 and 0.324 nm could be ascribed to the (101) crystal plane of anatase TiO_2_ (A‐TiO_2_) and (110) plane of rutile TiO_2_ (R‐TiO_2_), respectively (Figure 1d).^[^ ^31^ ^]^ The filtered image shows discontinuities in the TiO_2_ lattice stripes as well as blurring or even missing of some lattice positions, confirming the presence of some defects (Figure 1e). Interestingly, only the pattern for the TiO_2_ but no lattice spacing for the Ru species is observed due to overlapping lattice of Ru and TiO_2_.^[^ ^32^ ^]^ Further, the high‐angle annular dark‐field scanning TEM (HAADF‐STEM) image demonstrates the bright Ru islands in flat shape highlighted by the pink cycles are clearly visualized relative to the dark TiO_2_ background (Figure 1f), and Ru with ultra‐small sub‐nanoclusters of about 1 nm or so has penetrated and even grown along the (101) face of anatase TiO_2_ or the (110) face of rutile TiO_2_ (Figure 1f,g). This is strong evidence that Ru nanoclusters are confined into the lattice frames of TiO_2_, which favors a tensile strain effect occurred for the embedded Ru. Such lattice distortion can form more interfacial chemical bonds and then increase active sites for electrocatalysis.^[^ ^33^ ^]^ Then, we measured the local distance between a Ru atom and a nearby Ti atom by aberration‐corrected HAADF‐STEM image of F‐Ru@TiO_2_ (Figure 1h,i). The detected distance of 0.276 nm can be attributed to Ru–Ti interfacial bonds in that this distance is shorter than the sum of the atomic radii of Ti and Ru (0.296 nm) and no room can be left to insert O atom.^[^ ^24^ ^]^ However, the detected interfacial distance of 0.356 nm corresponds to the Ru–O–Ti bond due to the insertion of O atoms that leads to the space expansion between Ru and Ti. These results support the coexistence of Ru–Ti and Ru–O–Ti bonds in F‐Ru@TiO_2_. The EDS mappings of F‐Ru@TiO_2_ exhibit uniform distribution and overlapping of Ru, Ti, and O elements, superior to the overlapping of Ru and C elements (Figure 1j), confirming that the Ru nanoclusters are mainly anchored on the TiO_2_ support. And by comparison, it was found that HS‐Ru@TiO_2_ reference has noticeable hemispherical Ru clusters marked by pink line with relatively large Ru sizes (appropriately 2 nm), which is formed due to insufficient confinement of Ru within the TiO_2_ matrix (Figure S3, Supporting Information). It is reported that strengthened metal‐support interaction (MSI) would benefit to generating flat Ru structures instead of hemispherical shape.^[^ ^34^ ^]^ Hence, we can conclude that F‐Ru@TiO_2_ fabricated by the H_2_/Ar reduction strategy possesses stronger interfacial interaction and more chemical bonds between Ru and TiO_2_ than those obtained by the hydrothermal treatment.

**Figure 2:**
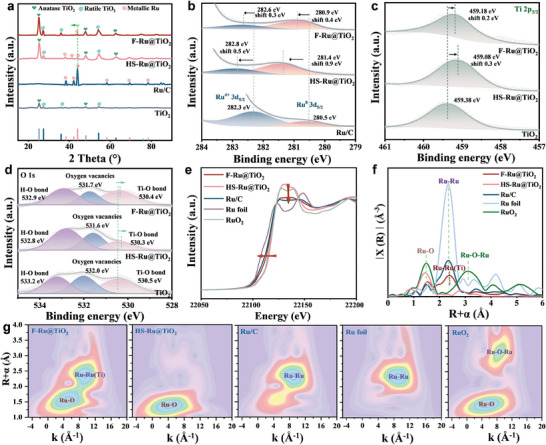
a) XRD patterns, b) Ru 3d XPS spectra, c) Ti 2p XPS spectra, and d) O 1s XPS spectra of F‐Ru@TiO_2_, HS‐Ru@TiO_2_, Ru/C and TiO_2_ electrocatalysts. e) Ru K‐edge XANES curves, f) EXAFS spectra, and g) WT‐EXAFS plots of F‐Ru@TiO_2_, HS‐Ru@TiO_2_, and Ru/C with Ru foil and RuO_2_ as references.

The crystal structure of electrocatalysts is analyzed by X‐ray diffraction (XRD, **Figure** **2**a), and the coexistence of anatase TiO_2_ (PDF#00‐021‐1272), rutile TiO_2_ (PDF#00‐021‐1276) and hexagonal close‐packed Ru (hcp Ru, PDF#04‐001‐1921) in F‐Ru@TiO_2_ and HS‐Ru@TiO_2_ is observed. Compared with Ru/C (Figure S4, Supporting Information), the diffraction peak intensity assigned to hcp Ru in F‐Ru@TiO_2_ and HS‐Ru@TiO_2_ is obviously smaller. In additon, F‐Ru@TiO_2_ displays the broader diffraction peaks for Ru phases in contrast to HS‐Ru@TiO_2_, which means that MSI is more enhanced in the following order Ru/C < HS‐Ru@TiO_2_ < F‐Ru@TiO_2_. Careful observation that the local XRD peaks of Ru in F‐Ru@TiO_2_ and HS‐Ru@TiO_2_ shift to the lower degree. This suggests that the tensile strains are significantly included in F‐Ru@TiO_2_ and HS‐Ru@TiO_2_, which are brought by the lattice mismatch of metal Ru and TiO_2_ during stabilization of Ru into TiO_2_ frames. Such unique stress effects may endow the F‐Ru@TiO_2_ with improved electrocatalytic properties.^[^ ^35^ ^]^ X‐ray photoelectron spectroscopy (XPS) was further explored to examine the electronic properties of as‐prepared Ru‐based materials. As shown in Figure 2b, the binding energy of Ru^0^ 3d_5/2_ peak of F‐Ru@TiO_2_ and HS‐Ru@TiO_2_ positively shifts by 0.4 and 0.9 eV relative to Ru/C, respectively. At the same time, their Ti 2p_3/2_ and O 1s spectra show obvious negative‐shift after interacting with the Ru metal (Figure 2c,d). It is certain that the presence of heterogeneous interface is benefical for creating apparent electron‐deficient state on the Ru surface. Notably, although F‐Ru@TiO_2_ shows the strongest MSI, its binding energy of Ru^0^ 3d_5/2_ is located between Ru/C and HS‐Ru@TiO_2_, and is very close to those of Ru/C, which seems unusual. In other words, electron‐deficient trends on the Ru surface (Ru/C < F‐Ru@TiO_2_ < HS‐Ru@TiO_2_) is inconsistent with the increased MSI (Ru/C < HS‐Ru@TiO_2_ < F‐Ru@TiO_2_). We infer that interfacial chemistry bonds on F‐Ru@TiO_2_ and HS‐Ru@TiO_2_ may be different. To make interfacial environment in the electrocatalysts more clearer, the electron paramagnetic resonance (EPR) spectra were performed (Figure S5, Supporting Information). It is seen that pure and original TiO_2_ contains an intensive EPR signal of oxygen vacancies at *g* = 2.003,^[^ ^36^ ^]^ which would provide more trapping sites to confine Ru. After inserting Ru with similar loading, existing oxygen vacancies attenuate and the oxygen vacancies of F‐Ru@TiO_2_ are even lower than that of HS‐Ru@TiO_2_, which reveals that Ru atoms of the former occupy more oxygen vacancies and own more chemical bonds at the interface. Consistently, F‐Ru@TiO_2_ confines Ru nanoclusters in flat shape, rather than those in hemispherical shape on HS‐Ru@TiO_2_. Additionally, previous reports revealed that H_2_ annealing could greatly facilitate the co‐reduction of Ru and Ti to generate Ru–Ti bond with the presence of Ru–O–Ti bonds,^[^ ^37^ ^]^ which is in alignment with our findings derived from aberration‐corrected HAADF‐STEM that Ru–Ti and Ru–O–Ti bonds coexist in F‐Ru@TiO_2_. Given more interfacial chemical bonds for F‐Ru@TiO_2_, we also reasonably speculate that HS‐Ru@TiO_2_ only contains Ru–O–Ti bonds due to the lack of H_2_ annealing process and a weaker MSI.

To further confirm the above point, X‐ray absorption spectroscopy (XAS) is employed to determine the electronic structures and coordination environments of F‐Ru@TiO_2_ and HS‐Ru@TiO_2_ electrocatalysts.^[^ ^38^ ^]^ X‐ray absorption near‐edge structure (XANES) analysis exhibits that the white line intensity of Ru atom features in F‐Ru@TiO_2_ are similar to those in Ru/C, while HS‐Ru@TiO_2_ is closer to RuO_2_ reference, illustrating that Ru in F‐Ru@TiO_2_ and Ru/C has low oxidation state compared with HS‐Ru@TiO_2_ (Figure 2e).^[^ ^39^ ^]^ Moreover, the adsorption edge of Ru K‐edge moves toward the higher energy and follows the trend of Ru/C < F‐Ru@TiO_2_ < HS‐Ru@TiO_2_, suggesting that Ru donates electrons to interfacial atoms and the *d‐*bands electron of the confined Ru nanoclusters is deficient, which is in good agreement with the XPS result. The Ru K‐edge Fourier‐transformed extend X‐ray absorption fine structure (FT‐EXAFS) curves are depicted in Figure 2f. The Ru foil reference presents the prominent peak of Ru–Ru bond at 2.35 Å, and RuO_2_ reference shows two peaks corresponding to the Ru–O coordination (≈1.51 Å) and Ru–O–Ru coordination (≈3.18 Å).^[^ ^40^ ^]^ As expected, F‐Ru@TiO_2_ possesses two peaks associating with Ru–O bond at 1.51 Å and Ru–Ru (Ti) bond at 2.40 Å because bond strengths of Ru–Ru and Ru–Ti are similar.^[^ ^41^ ^]^ The positive shifts of the weak Ru–Ru (Ti) peak of F‐Ru@TiO_2_ compared with that of Ru foil is due to weak coordination number (CN) and the lattice mismatch of confined Ru and TiO_2_. Furthermore, there is no peak belonging to Ru–O–Ru at 3.18 Å, verifying the formation of Ru–O–Ti bonds for F‐Ru@TiO_2_.^[^ ^42^ ^]^ In contrast, HS‐Ru@TiO_2_ (Figure 2f) only displays one peak at 1.51 Å (Ru–O bond) but the disappearance of Ru–Ru (Ti) bond (2.40 Å) and Ru–O–Ru bond (3.18 Å), clearly indicating that in HS‐Ru@TiO_2_, the existence of Ru–O–Ti is dominant and there is no Ru–Ti coordination. Also, the fitting of Ru K‐edge EXAFS is presented in Figure S6 and Table S1 (Supporting Information). The F‐Ru@TiO_2_ exhibits the lower coordination number (CN, 1.4 ± 0.3) and the longer bond length of Ru–Ru (Ti) (2.70 ± 0.01 Å) compared with those of Ru foil (CN: 12, bond length: 2.68 ± 0.01 Å), further revealing their untrafine Ru diameter with tensile strain. In the intuitively executed wavelet transform (WT) (Figure 2g), absent Ru–O–Ru signal and present Ru–O and Ru–Ru (Ti) signals are observed in F‐Ru@TiO_2_, whereas the WT of HS‐Ru@TiO_2_ and Ru foil only show the existence of Ru–O signal and Ru–Ru signal, respectively.^[^ ^14^ ^]^ Combining the aberration‐corrected HAADF‐STEM, EPR, and XAS results, we corroborate the enhanced interactions between Ru and TiO_2_ on F‐Ru@TiO_2_ and HS‐Ru@TiO_2_. Moreover, F‐Ru@TiO_2_ possesses coexisting interfacial Ru–Ti and Ru–O–Ti bonds, while HS‐Ru@TiO_2_ only contains interfacial Ru–O–Ti bonds. Then, the above‐mentioned phenomenon that the valence of Ru in F‐Ru@TiO_2_ is lower than that of HS‐Ru@TiO_2_ can be reasonably attributed to present electron transfer from Ti (electronegativity value of Ti, χ_Ti_ = 1.54) to Ru (χ_Ru_ = 2.20) via Ru–Ti bonds for the former. The apparent valences of Ru in F‐Ru@TiO_2_ and HS‐Ru@TiO_2_ are still higher than that of Ru/C, arising from the contribution of their Ru–O–Ti coordinations and the substantial electron migration from Ru atoms to O atoms (χ_O_ = 3.44). These lay the foundation for studying the effect of interfacial chemical bonds on electrocatalytic performance.

### 2.2 Electrochemical Performance toward Alkaline HER

**Figure 3:**
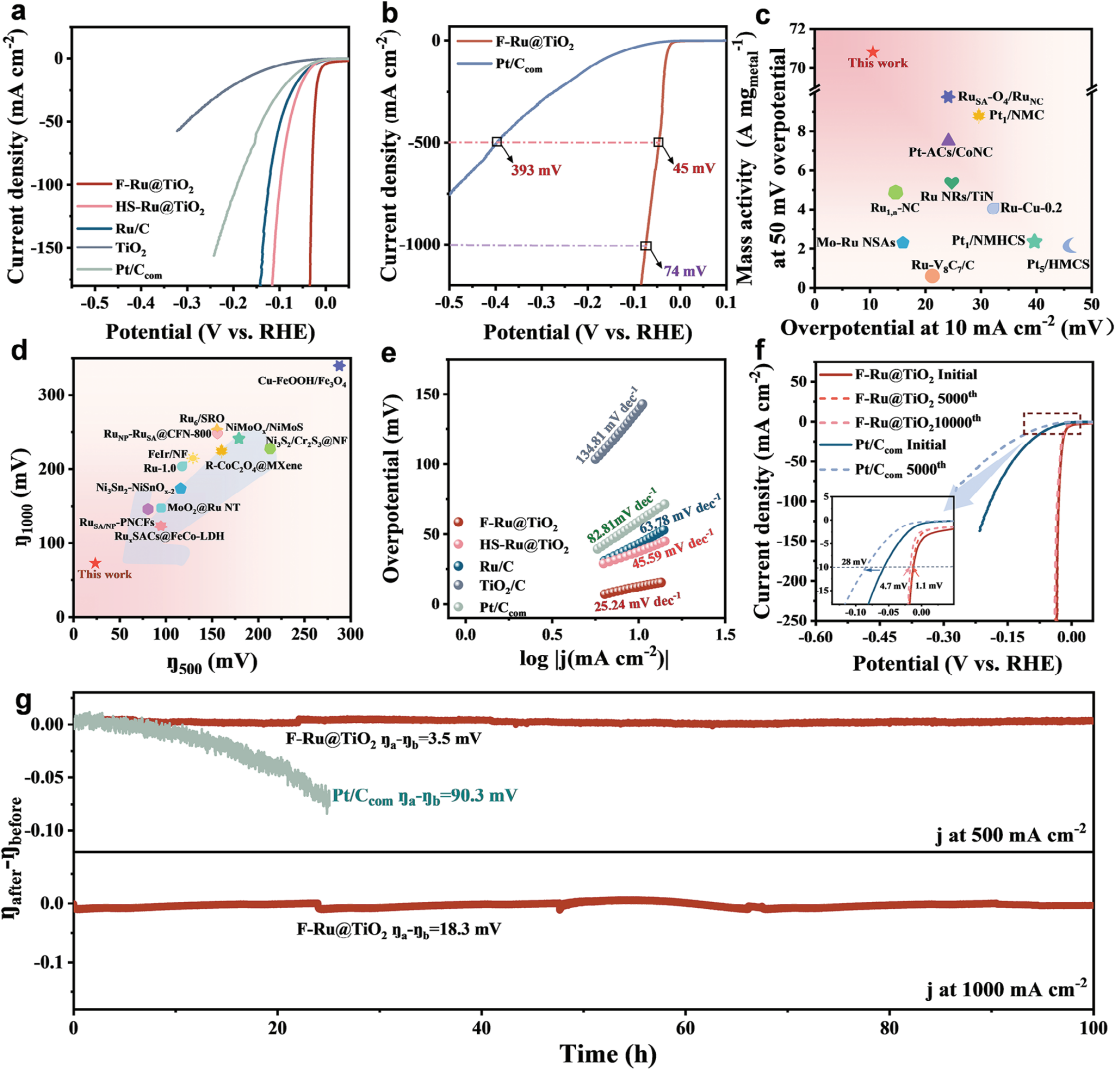
a) HER polarization curves of F‐Ru@TiO_2_, HS‐Ru@TiO_2_, Ru/C, and Pt/C electrocatalysts in 1.0 m KOH. b) HER polarization curves of F‐Ru@TiO_2_ and Pt/C over a wide current density in 1.0 m KOH. c) Comparison of the HER MA at 50 mV overpotential and overpotentials at 10 mA cm^−2^ of F‐Ru@TiO_2_ with recently reported HER electrocatalysts. d) Comparison of overpotentials at 500 mA cm^−2^ and 1000 mA cm^−2^ for the recently reported HER electrocatalysts. e) Tafel plots extracted from (a). f) HER polarization curves of F‐Ru@TiO_2_ and Pt/C before and after CV cycles. g) Long‐term stability tests of F‐Ru@TiO_2_ at 500 and 1000 mA cm^−2^ for 100 h, with Pt/C serving as a comparative benchmark at a current density of 500 mA cm^−2^ for a duration of 25 h.

The electrocatalytic property with respect to the alkaline HER was first evaluated in 1.0 m KOH. As shown in **Figure** **3**a, the electrocatalytic activity decreases in the following order F‐Ru@TiO_2_ > HS‐Ru@TiO_2_ > Ru/C, and the F‐Ru@TiO_2_ exhibits the best performance, where the overpotential (η) just requires 12 mV to reach the current density of 10 mA cm^−2^, while higher overpotentials are needed for HS‐Ru@TiO_2_ of η_10_ = 38 mV, Ru/C of η_10_ = 43 mV and Pt/C of η_10_ = 57 mV. It implies that constructed Ru–O–Ti bond at the interface is beneficial to promote H_2_ production, and the coexistence and synergism of Ru–Ti and Ru–O–Ti interfacial bonds is more favorable for the enhancement of electrocatalytic H_2_ evolution (Figure S7, Supporting Information). When the current density is increased to 500 mA cm^−2^, the overpotential of F‐Ru@TiO_2_ is only 45 mV (Figure 3b), much smaller than the value of Pt/C (393 mV). After that, the mass activity (MA) normalized with the mass loading of noble metals is estimated in Figure 3c. F‐Ru@TiO_2_ corresponds to an ultrahigh MA value of 70.6 A mg_Ru_^−1^ at 50 mV overpotential. With such low overpotential and high MA (Figure 3c, Tables S2 and S3, Supporting Information), F‐Ru@TiO_2_ is a superior intrinsic H_2_ production electrocatalyst in alkaline media. Moreover, a high current density of 1000 mA cm^−2^ is attainable at an astonishingly low overpotential of merely 74 mV (Figure 3d). Tafel plots further reveal the accelerated HER kinetics, in which F‐Ru@TiO_2_ pocesses the lowest Tafel slope of 25.24 mV dec^−1^, in comparison with HS‐Ru@TiO_2_ (45.59 mV dec^−1^), Ru/C (63.78 mV dec^−1^), and 20% Pt/C (82.81 mV dec^−1^, Figure 3e). Such a small Tafel value on F‐Ru@TiO_2_ suggests that the Volmer–Tafel reaction mechanism is followed and the chemical desorption of H_ad_ is the rate‐determining step for the HER process.^[^ ^43^ ^]^ In the electrochemical impedance spectroscopy (EIS) study, F‐Ru@TiO_2_ exhibits the lowest charge transfer resistance (R_ct_), at merely 7.4 Ω (Figure S8a, Supporting Information), which suggests the accelerated charge transfer rate and thus enhanced HER kinetics. Consistently, the turnover frequency (TOF) of F‐Ru@TiO_2_ achieves a remarkable 37.04 s^−1^ at 50 mV, outperforming HS‐Ru@TiO_2_, Ru/C, and 20% Pt/C by factors of 36.7, 49.4, and 82.3, respectively (Figure S8b, Supporting Information).

Durability is another key parameter in evaluating a HER electrocatalyst. After 5000 or 10 000 cycles of CV, no obvious decay is observed for F‐Ru@TiO_2_ according to the LSV curves (Figure 3f). However, Pt/C experiences a serious attenuation after only 5000 cycles of CV tests. Further, the F‐Ru@TiO_2_ is subjected to chronopotentiometry test at a current density of 500 mA cm^−2^ and only a 3.5 mV increase of overpotential is exhibited after continuous operation for 100 h. This performance significantly surpasses that of the Pt/C electrocatalyst, which shows a dramatic overpotential increase of 90.3 mV after just 25 h of operation (Figure 3g). Given that F‐Ru@TiO_2_ can achieve a large current density at a relatively low overpotential, its long‐term stability was further evaluated under the industry‐level large current density of 1000 mA cm^−2^. As illustrated in Figure 3g, an insignificant increase of just 18.3 mV in overpotential is observed after an extended 100 h chronopotentiometry test, underscoring its potential as an exceptional HER electrocatalyst for industrial application. Post‐reaction characterizations of TEM and HR‐TEM clearly exhibit that two kinds of crystal phases (anatase and rutile TiO_2_) are also distinguished on the F‐Ru@TiO_2_ electrocatalyst (Figure S9a,b, Supporting Information). The EDS elemental mapping reveals a uniform distribution of Ru throughout the entire substrate material (Figure S9c,d, Supporting Information). XRD patterns and XPS demonstrate the well‐preserved crystalline structure and active Ru component in F‐Ru@TiO_2_ (Figure S9e–g, Supporting Information). In contrast, commercial Pt/C undergoes an obvious aggregation and visible size change after durability (Figure S10, Supporting Information). These results provide compelling evidence of excellent durability of F‐Ru@TiO_2_ in HER.

### 2.3 Electrochemical Performance toward Alkaline HOR

**Figure 4:**
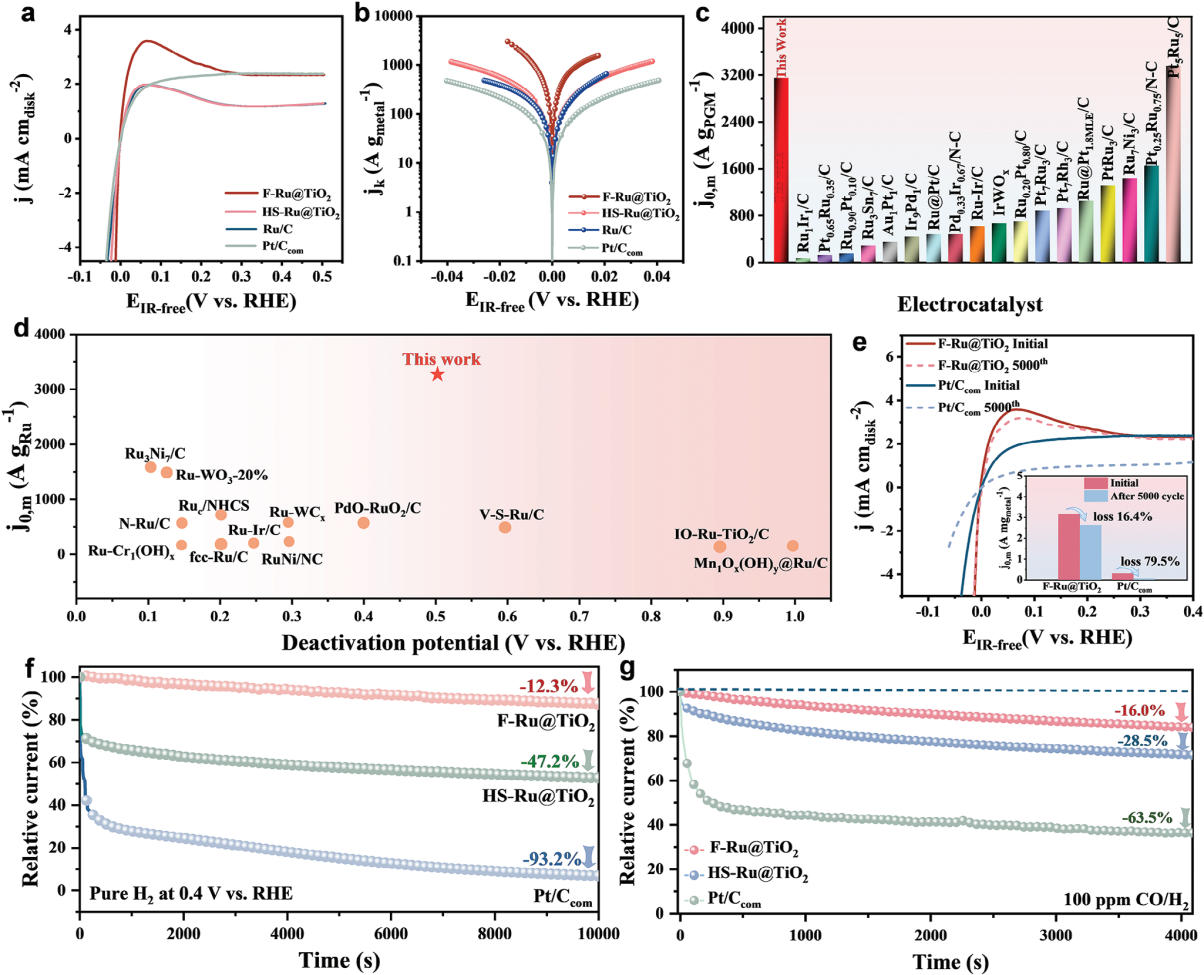
a) HOR polarization curves and b) HOR Tafel plots of F‐Ru@TiO_2_, HS‐Ru@TiO_2_, Ru/C and Pt/C electrocatalysts in H_2_‐saturated 0.1 m KOH. c) Comparison of j_0,m_ of F‐Ru@TiO_2_ with recently reported Ru‐based HOR electrocatalysts. d) Continuous potentials and j_0,m_ of F‐Ru@TiO_2_ and reported Ru‐based electrocatalysts for the alkaline HOR. e) HOR polarization curves of F‐Ru@TiO_2_ and Pt/C before and after CV cycles. f) Chronoamperometry tests of F‐Ru@TiO_2_, HS‐Ru@TiO_2_, and Pt/C at 0.4 V vs. RHE for 10 000 s in H_2_‐saturated 0.1 m KOH electrolyte. g) Chronoamperometry tests of F‐Ru@TiO_2_, HS‐Ru@TiO_2_, and Pt/C at 0.1 V vs. RHE for 4000 s in 100 ppm CO/H_2_‐saturated 0.1 m KOH electrolyte.

Then, we examine whether the crystalline lattice‐confined Ru in TiO_2_ can also show distinctive alkaline HOR behaviors. As illustrated in **Figure** **4**a, it is apparent that F‐Ru@TiO_2_ achieves the highest anodic current density at the kinetic control region and the activity has the order of F‐Ru@TiO_2_ > HS‐Ru@TiO_2_ > Ru/C. Notably, the anodic current density of F‐Ru@TiO_2_ is comparable with the state‐of‐the‐art Pt/C even up to the potential of 0.5 V vs. RHE. For a large power output under real work conditions, a HOR electrocatalyst should maintain higher activity up to a potential of 0.3 V vs. RHE.^[^ ^25^ ^]^ However, HOR/HER current densities of HS‐Ru@TiO_2_, Ru/C, and other reported Ru‐based electrocatalysts are inferior to that of Pt/C when the potential is above 0.15 V vs. RHE because of an excessive combination of Ru with the O species.^[^ ^44^, ^45^ ^]^ Therefore, it can be concluded that both the Ru–Ti and Ru–O–Ti interfacial chemical bonds are irreplaceable in the alkaline HOR activity, and Ru–Ti bonds play absolutely a crucial role in the catalytic HOR performance at the high potential for F‐Ru@TiO_2_. Additionally, the kinetic current density (j_k_) was extracted from the Koutecky–Levich equation, and it was further normalized by the respective loading of noble metals to obtain the mass‐specific kinetic current density (j_k,m_). Mass‐specific exchange current density (j_0,m_) was calculated via fitting j_k,m_ according to the Butler–Volmer equation (Figure 4b).^[^ ^46^ ^]^ The F‐Ru@TiO_2_ presents an amazing j_0,m_ of 3155 A g_Ru_^−1^, which is about 4.4‐, 4.8‐, and 10.1‐fold those of the HS‐Ru@TiO_2_, Ru/C, and Pt/C counterparts, respectively. To our best knowledge, the j_0,m_ of F‐Ru@TiO_2_ surpasses that of most known Ru‐based HOR electrocatalysts in the alkaline media (Figure 4c) and its potential stability ranks at the top in the literature (Figure 4d; Table S4, Supporting Information). Furthermore, the LSV polarization curves were obtained at various rotating speeds (as shown in Figure S11, Supporting Information). By plotting the inverse of current density (j^−1^) at a potential of 0.1 V vs. RHE against the square root of the inverse of rotating speed (ω^−1/2^), a linear relationship is established. The slopes of the fitted line for F‐Ru@TiO_2_ and Pt/C are determined to be 4.58 and 4.16 cm^2^ mA^−1^ s^−1/2^, respectively, which are in close proximity to the theoretical value of 4.87 cm^2^ mA^−1^ s^−1/2^, indicative of a two‐electron HOR process.

Besides the outstanding activity, the dynamic HOR stability of F‐Ru@TiO_2_ was also measured through CV (Figure 4e). The HOR polarization curve of F‐Ru@TiO_2_ after 5000 potential cycles almost overlaps with the initial one, retaining 83.6% of the original current density, which is higher than that of Pt/C (20.5%). To demonstrate the HOR anti‐deactivation at higher potentials, we performed tests at 0.4 V vs. RHE. As seen from the chronoamperometry test results, the F‐Ru@TiO_2_ electrocatalyst displays excellent anti‐deactivation features for alkaline HOR, where it experiences a current drop of 12.3% at even 0.4 V vs. RHE over 10 000 s (Figure 4f). This is much less severe than exhibited by the HS‐Ru@TiO_2_ (47.2% loss) and commercial Pt/C (93.2% loss). TEM, XRD and XPS of the aged F‐Ru@TiO_2_ demonstrated that the F‐Ru@TiO_2_ electrocatalyst owns stable morphology, phase, and electronic behavior in the HOR process, indicative of the material's promise for the practical fuel‐cell application (Figure S12, Supporting Information).

It is known that CO poisoning is another intractable problem for anodic HOR electrocatalysts since current H_2_ is mainly supplied by reforming fossil fuels containing CO impurity.^[^ ^47^ ^]^ Subsequently, chronoamperometry tests at 𝜂 = 0.1 V vs. RHE for 4000 s in 100 ppm CO/H_2_‐saturated 0.1 m KOH electrolyte were conducted to inspect their CO tolerance. As displayed in Figure 4g, the current density of F‐Ru@TiO_2_ only decreases by 16.0%, while the HS‐Ru@TiO_2_ and Pt/C show severe 28.5% and 63.5% deteriorations. Furthermore, the TEM and HRTEM images reveal no notable variations in the structure of the aged F‐Ru@TiO_2_ after the CO poisoning test (Figure S13, Supporting Information), suggesting the superior CO tolerance ability of F‐Ru@TiO_2_. The comparison of the HOR stability and anti‐CO toxicity of F‐Ru@TiO_2_ with reported Ru‐based electrocatalysts are summarized in Tables S4 and S5 (Supporting Information), confirming that F‐Ru@TiO_2_ is the most advanced elctrocatalyst for hydrogen energy conversion.

### 2.4 Understanding the Role of Interfacial Chemical Bonds

**Figure 5:**
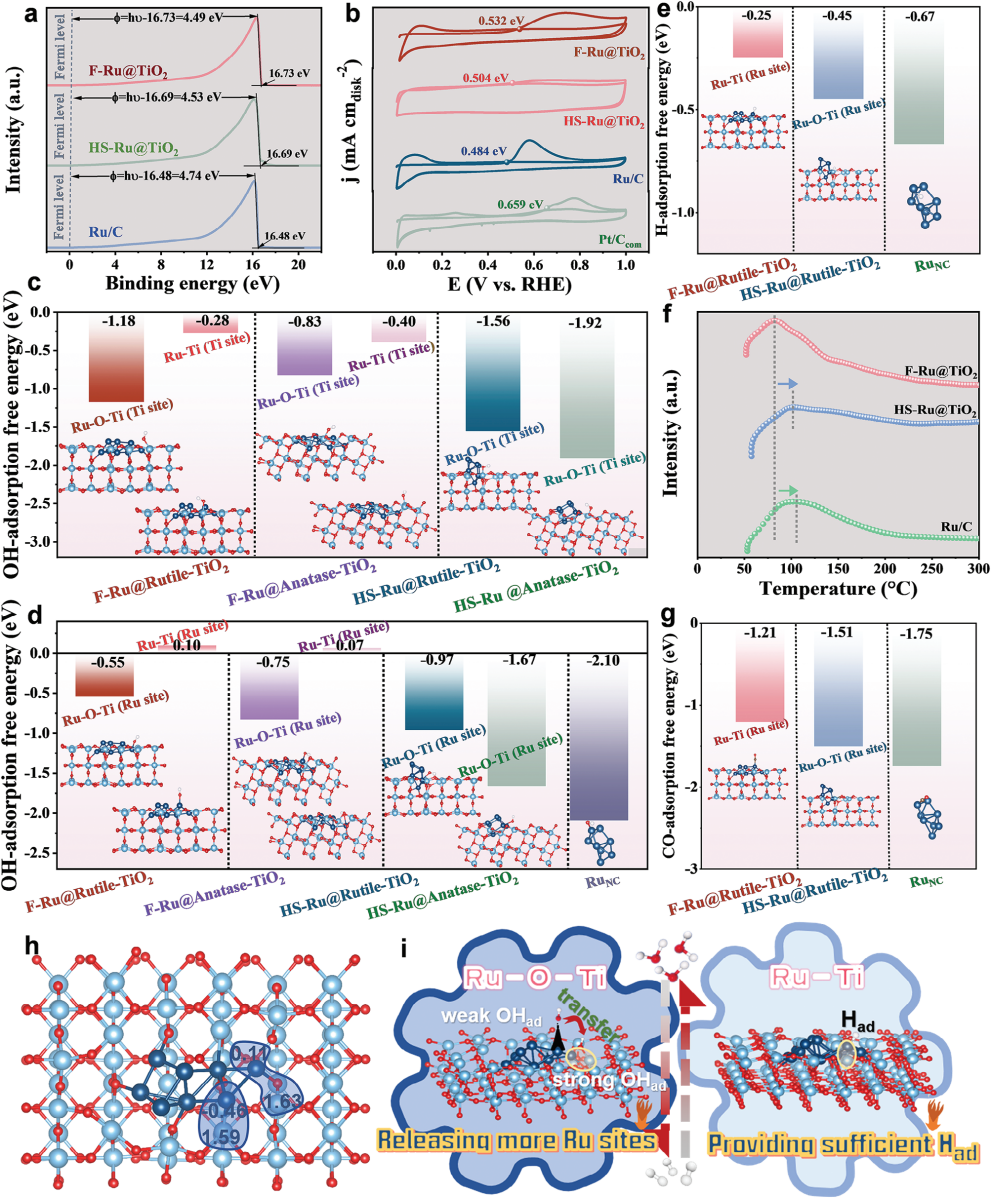
a) UPS spectra of F‐Ru@TiO_2_, HS‐Ru@TiO_2_ and Ru/C. b) CO stripping curves of F‐Ru@TiO_2_, HS‐Ru@TiO_2_, Ru/C and Pt/C. c) Adsorption free energy of OH_ad_ intermediate on the F‐Ru@TiO_2_ (Ti, Ru–O–Ti), F‐Ru@TiO_2_ (Ti, Ru–Ti), HS‐Ru@TiO_2_ (Ti, Ru–O–Ti). d) Adsorption free energy of OH_ad_ intermediate on the F‐Ru@TiO_2_ (Ru, Ru–O–Ti), F‐Ru@TiO_2_ (Ru, Ru–Ti), HS‐Ru@TiO_2_ (Ru, Ru–O–Ti), and Ru. e) Adsorption free energy of H_ad_ intermediate on the F‐Ru@TiO_2_ (Ru, Ru–Ti), HS‐Ru@TiO_2_ (Ru, Ru–O–Ti) and Ru. f) H_2_‐TPD of F‐Ru@TiO_2_, HS‐Ru@TiO_2_ and Ru/C. g) Adsorption free energy of CO_ad_ intermediate on the F‐Ru@TiO_2_ (Ru, Ru–Ti), HS‐Ru@TiO_2_ (Ru, Ru–O–Ti) and Ru. h) The Bader charge of F‐Ru@TiO_2_. i) The synergistic effect of the Ru–O–Ti and Ru–Ti bonds.

From the above experiments, the Ru–Ti and Ru–O–Ti interfacial chemical bonds in F‐Ru@TiO_2_ can drastically improve the HER/HOR activity, and Ru–Ti is a dominant influence factor responding to a significant HOR current density at high potential. Then, we explore why Ru–Ti and Ru–O–Ti interfacial chemical bonds can be of benefit to hydrogen energy conversion and what is the role of Ru–Ti in achieving a comparable HOR current density to that of Pt/C, particularly up to a potential of 0.5 V vs. RHE. Specific adsorption behavior of oxygen‐containing species was first observed by ultraviolet photoelectron spectroscopy (UPS) and CO‐stripping experiment.^[^ ^48^ ^]^ Remarkably, the measured work functions (WFs) decrease in the order Ru/C > HS‐Ru@TiO_2_ > F‐Ru@TiO_2_ (**Figure** **5**a). The WF of F‐Ru@TiO_2_ (4.49 eV) is 0.04 and 0.25 eV lower than those of HS‐Ru@TiO_2_ (4.53 eV) and Ru (4.74 eV), respectively, indicating more disordered interfacial water molecule network and thus the weaker adsorption of OH_ad_ on F‐Ru@TiO_2_.^[^ ^49^, ^50^ ^]^ Similar to the UPS results, the starting potential of the CO stripping curve on F‐Ru@TiO_2_ (0.532 V vs. RHE) is positive compared with HS‐Ru@TiO_2_ (0.504 V vs. RHE) and Ru/C (0.484 V vs. RHE, Figure 5b), implying that easy desorption of OH intermediates on Ru sites in F‐Ru@TiO_2_.^[^ ^51^ ^]^ Impressively, the detraction of OH binding energy on the F‐Ru@TiO_2_ does not lead to overly weak OH adsorption in that the starting potential of the CO_ad_ stripping curve on F‐Ru@TiO_2_ shifts negatively as compared to Pt/C (0.659 V vs. RHE), OH adsorption‐desorption on the F‐Ru@TiO_2_ has been well balanced.^[^ ^52^ ^]^

DFT calculations were employed to further get insights into the OH active sites and elaborate the alterations in its adsorption energy by taking TiO_2_ crystal confined Ru. Firstly, we constructed the F‐Ru@TiO_2_ models with flat Ru clusters embedded into anatase and rutile TiO_2_ according to the experimental results, and the inserted Ru clusters in hemispherical shape (HS‐Ru@TiO_2_), as well as pure Ru clusters models (Figures S14–S16, Supporting Information), were also constructed as a comparison. As seen in Figure 5c and Figures S17–S20 (Supporting Information), OH_ad_ tends to preferentially adsorb on the Ti site of Ru–O–Ti interfacial chemical bonds, which can greatly liberate active Ru atoms and protect Ru sites from passivation by OH_ad_ blocking.^[^ ^53^ ^]^ As expected, different Ru sites in F‐Ru@TiO_2_ also exhibit much higher OH adsorption energy than those of HS‐Ru@TiO_2_ and pure Ru clusters, again indicative of the better OH desorption kinetics of Ru sites in F‐Ru@TiO_2_ (Figure 5d). The value of OH‐adsorption free energy (ΔG_OH*_) of Ru sites in Ru–Ti bonds is even 0.10 or 0.07 eV, which is unfavorable for binding OH_ad_ (Figure 5d), providing full prerequisites for the adsorption‐desorption of crucial H_ad_ intermediates.^[^ ^54^ ^]^ Subsequently, the H‐adsorption free energy (Δ*G*_H*_) is achieved.^[^ ^55^ ^]^ Released Ru sites, especially for Ru in interfacial Ru–Ti bonds, process a near‐zero Δ*G*_H*_ value (−0.25 eV, Figure 5e; Figures S21a and S22b, Supporting Information). The optimized hydrogen binding strength (HBE) can be also validated by hydrogen‐temperature programmed desorption (H_2_‐TPD). F‐Ru@TiO_2_ corresponds to the lowest desorption temperature (Figure 5f), consistent with the reduced HBE. In addition, compared with those on HS‐Ru@TiO_2_ and pure Ru clusters (Figures S21b and S22c, Supporting Information), the adsorption of CO on F‐Ru@TiO_2_ is also significantly weakened (Figure 5g), suggestive of improved resistance to CO.^[^ ^56^ ^]^ Therefore, our theoretical calculation demonstrates that the OH_ad_ easily approaches the Ti sites of Ru–O–Ti interfacial chemical bonds for F‐Ru@TiO_2_, preventing the over‐adsorption of OH_ad_ on all Ru sites. These Ru sites, especially for Ru in Ru–Ti bonds with unfavorable adsorption of OH_ad_, are responsible for the provision of required H_ad_. The synergistic boosting of the OH_ad_ transfer and H_ad_ supply process on the Ru–O–Ti and Ru–Ti bonds, respectively, is attributed to the enhancement of the HOR/HER activity, stability, and a comparable HOR current density to that of Pt/C at 0.5 V vs. RHE.

Such an outcome could be directly related to the unique electronic structure of F‐Ru@TiO_2_.^[^ ^57^ ^]^ The Bader charge analysis displays that Ti site in Ru–O–Ti interfacial chemical bonds has a pronounced electron‐deficient nature (+1.63 e, Figure 5h). Positively charged Ti would readily interact with the lone pair electrons of the oxygen in OH_ad_ via Lewis acid‐base pairing, in line with the ΔG_OH*_ results that the Ti site in Ru–O–Ti can efficiently capture OH_ad_.^[^ ^58^ ^]^ However, the electron‐enrichment on the Ru site in Ru–Ti interfacial chemical bonds (‐0.46 e) would have an unfavorable affinity towards oxygen‐containing species because of the electrostatic repulsion between surface electrons and negative‐charged O nature.^[^ ^59^ ^]^ This makes the H‐species molecule activated and easier to exclusively form at Ru–Ti interfaces. Thus, we conclude that well‐altered OH binding on F‐Ru@TiO_2_ is conducive to H adsorption due to the coexistence of Ru–O–Ti and Ru–Ti bonds (Figure 5i), leading to a wide potential‐stable window toward alkaline HOR and a low overpotential at industry‐level large current density for alkaline HER.

## 3 Conclusion

In summary, we have demonstrated that partial Ru phases inserted into metal‐oxide electrocatalysts are actually unacceptable under the industry‐level large HER current density or the elevated anodic HOR potential due to the unsatisfactory regulation of single‐bond such as Ru–O–Ti interfacial chemical bond. Thus, we completely confine Ru into the lattice framework of TiO_2_ to generate Ru clusters in flat shape (F‐Ru@TiO_2_) through H_2_ annealing, in which H_2_ accelerates the bond cleavage and the escape of O atoms to form the coexisting Ru–Ti and Ru–O–Ti bonds. Ti sites on Ru–O–Ti bonds facilitate the OH adsorption and the interaction of OH_ad_ and Ru is not very strong. Especially in Ru–Ti bonds, Ru is not conducive to OH adsorption, which is beneficial to exclusive H_ad_ supply. As predicted, the achieved F‐Ru@TiO_2_ possesses an ultralow HER overpotential of 74 mV at 1000 mA cm^−2^ and extremely high HOR activity of 3155 A g_Ru_^−1^. Besides, F‐Ru@TiO_2_ exhibits a negligible performance decay after 100 h operation at large HER current density of 1000 mA cm^−2^ and maintains stable HOR electrocatalytic activity at high potential of 0.5 V vs. RHE, corroborating the F‐Ru@TiO_2_ is a promising electrocatalyst in hydrogen storage and conversion system.

## Conflict of Interest

The authors declare no conflict of interest.

## Supporting information



Supporting Information

## Data Availability

The data that support the findings of this study are available from the corresponding author upon reasonable request.
