# Supplementary material for: Confining Flat Ru Islands into TiO2 Lattice with the Coexisting Ru–O–Ti and Ru–Ti Bonds for Ultra‐Stable Hydrogen Evolution at Amperometric Current Density and Hydrogen Oxidation at High Potential
Source: Adv Sci (Weinh). 2024 Oct 25;11(48):2410881. doi: 10.1002/advs.202410881 (PMC11672276; doi:10.1002/advs.202410881)
Supplement: Supplementary file 1 — Supporting Information [file ADVS-11-2410881-s001.docx]

Supporting Information

**Confining Flat Ru Islands into TiO_2_ Lattice with the Coexisting Ru-O-Ti and Ru-Ti Bonds for Ultra-Stable Hydrogen Evolution at Amperometric Current Density and Hydrogen Oxidation at High Potential**

*Luyun Chen, Chunlei Li, Mengling Liu, Ziruo Dai, Haibin Wang, Xuan Zhou, Qiuping Zhao, and Yuanyuan Cong**

L. Chen, C. Li, M. Liu, Z. Dai, H. Wang, X. Zhou, Q. Zhao, Y. Cong
School of Petrochemical Technology
Lanzhou University of Technology
Gansu, Lanzhou 730050, China
E-mail: congyuanyuan@lut.edu.cn

L. Chen, C. Li, M. Liu, Z. Dai, H. Wang, X. Zhou, Q. Zhao, Y. Cong
Key Laboratory of Low Carbon Energy and Chemical Engineering of Gansu Province
Lanzhou University of Technology
Gansu, Lanzhou 730050, China

Content

A. Supplementary Experimental Section..........................…....………………….3

SI | Materials……….....................................................………………………3

SII | Electrocatalyst Preparation....................................................................3

SIII | Electrocatalyst Characterization….......................………………………4

SIV | Electrochemical Tests………..................................……………………5

SV | DFT Calculations……….......…......…......…...………………………...6

SVI | Activity Calculations of Alkaline HER....................................................7

SVII | Activity Calculations of Alkaline HOR....…..........…...........………….7

B. Supplementary Figures……….....….......…......….......…………….…..…8-26

C. Supplementary Tables…………..…..........................………………..…..27-32

D. Supplementary References.....…………….....…………............……………33

**A. Supplementary Experimental Procedures**

**SI | Materials.** Ruthenium (III) chloride hydrate (RuCl_3_·H_2_O, purity≥ 98.0%), N,N-dimethylformamide (DMF, purity≥ 99.0%), terephthalic acid (BDC, purity≥ 99.0%), triethylamine (TEA, purity≥ 99.5%), Pluronic F127 (F127, purity≥ 99.0%) and tetrabutyl titanate (TPOT, purity≥ 99 %) were obtained from Macklin Biochemical Co., Ltd. (Shanghai, China). Zinc chloride (ZnCl_2_, purity≥ 97.0%) was purchased from Aladdin Biochemical Technology Co., Ltd. (Shanghai, China). N-dodecylamine (DDA, purity≥ 99.0%), potassium hydroxide (KOH, purity ≥ 85.0%), sodium hydroxide (NaOH, purity ≥ 96.0%), ethanol (purity ≥ 95.0%) and cyclohexane (purity ≥ 99.9%) were bought from Sinopharm Chemical Reagent Co., Ltd. (Shanghai, China). Commercial Pt/C (20 wt%) was received from TANAKA Precious Metals Co., Ltd. (Japan, TKK). Ultrapure water (18.2 MΩ•cm at 25 °C) was used throughout all electrochemical tests.

**SII | Electrocatalyst Preparation.**

*2.1. Synthesis of conductive carbon support*

First, benzene-1,4-dicarboxylic acid (BDC, 0.25 g) was added to a mixture solution, including N,N-dimethylformamide (DMF, 64 mL), ethanol (4 mL), and deionized water (4 mL). After the ultrasonication for 30 min, zinc chloride (ZnCl_2_, 0.21 g) was added and dissolved, followed by the injection of triethylamine (TEA, 1.6 mL). The above mixture was stirred for 8 h at 25 °C, and the resulting powder after centrifugation, washing and drying was denoted as Zn-MOF nanosheets (Zn-BDC). The Zn-BDC powder was then heated to 950 °C with a ramping rate of 5 °C min^–1^ under Ar and maintained for 3 h. The final conductive carbon support was collected by etching the heat-treated Zn-BDC with 0.5 M nitric acid (HNO_3_) for 12 h at room temperature, washing with deionized water and drying under vacuum overnight.

*2.2. Synthesis of oxygen vacancy-enriched TiO_2_ nanospheres*

N-dodecylamine (DDA, 0.5 g) and Pluronic F127 (0.9 g) were added to ethanol (100 mL) and deionized water (5 mL), sonicated for 30 min. Then, tetrabutyl titanate (0.75 mL, TBOT was injected and vigorously stirred for 1 h at room temperature. After aging of 24 h at room temperature, the resulting white product was collected by centrifugation, washed three times with ethanol/deionized water, and then dried in an oven at 60 ^o^C for 12 h. Oxygen vacancy-enriched TiO_2_ nanospheres were readily facribated by carbonizing the above white product in a Ar atmosphere at 700 °C for 4 h with a heating rate of 5 °C min^–1^.

*2.3. Synthesis of flat Ru islands embedded into the lattice of TiO_2_ electrocatalyst (F-Ru@TiO_2_)*

Oxygen vacancy-enriched TiO_2_ nanospheres (TiO_2_, 0.05 g) and ruthenium (III) chloride (RuCl_3_, 30 mg) were added into a round bottom flask containing 30 ml of deionized water and dispersed homogeneously by sonication of 30 min, then conductive carbon support (0.05 g) was added and sonicated for another 30 min. The black powder was obtained by evaporation solvent in an oil bath at 100 °C. The black powder was transferred to a tube furnace and heated to 500 °C under H_2_/Ar (10 % H_2_, 90 % Ar) atmosphere at a ramping rate of 2 °C min^–1^ for 2 h. Finally, the cooled product was the F-Ru@TiO_2_ electrocatalyst.

*2.4. Synthesis of hemispherical Ru islands embedded into the lattice of TiO_2_ electrocatalyst (HS-Ru@TiO_2_)*

HS-Ru@TiO_2_ electrocatalyst was synthesized by replacing the impregnation and thermal reduction of F-Ru@TiO_2_ with a hydrothermal treatment at 180 °C for 20 h.

*2.5. Synthesis of* *Ru/C and TiO_2_/C references*

Ru/C and TiO_2_/C references were synthesized in the same procedures as F-Ru@TiO_2_, while leaving out TiO_2_ and RuCl_3_, respectively.

**SIII | Electrocatalyst Characterization.** X-ray powder diffraction (XRD) patterns was conducted using a Rigaku D/max-2400 diffractometer with Cu Kα radiation (λ = 1.5418 Å) from a generator operated at 40 kV and 30 mA. Transmission electron microscopy (TEM), high angle annular dark field (HAADF)-scanning TEM (STEM) and energy dispersive X-ray spectrometry (EDS) were performed on a FEI Talos F200x. Aberration corrected HAADF-STEM images were obtained using a FEI TITAN G2. X-ray photoelectron spectroscopy (XPS) was carried out at the Spring-8 with Al Kα radiation (15 kV, 10.8 mA, *hν* = 1486.6 eV) under a working pressure of 7.1 × 10^–5^ Pa. Calibration was performed using the internal standard of carbon deposit C (1s) with a binding energy of 284.6 eV. The X-ray absorption spectra (XAS) at the Ru K-edge were conducted at the beamline BL14W of the National Synchrotron Radiation Research Center (NSRRC) at Shanghai. The storage rings of SSRC were operated at 3.5 GeV with a stable current of 200 mA. Using Si (111) double-crystal monochromator, the data collection were carried out in fluorescence mode using Lytle detector. Electron paramagnetic resonance (EPR) spectra were measured by Bruker EMXplus-6/1 spectrometer. H_2_-temperature programmed desorption (H_2_-TPD) studies were performed in a ChemStar Instruments u-tube quartz reactor. Ultraviolet photoelectron spectroscopy (UPS, Thermo ESCALAB 250XI) was used to test the work function (WF). The WF was obtained as follows. A He discharge lamp with the He (I) photo line (21.22 eV) was applied, and the high-binding energy secondary electron cutoff (*E*_cutoff_) and the energy gap (Δ*E*) between the valence band maximum (VBM) and the fermi level were extracted from the UPS spectra. The WF was calculated based on the following equation.

$\text{WF}\text{ =}\text{ }\text{21.22}- \text{E}_{\text{cuttoff}}+ \Delta\text{E}\text{ }$ (Equation S1)

**SIV | Electrochemical Tests.** Electrochemical experiments were performed on a CHI-760 potentiostat with a three-electrode system (Pine Research Instruments). The working electrode and the counter electrode were a glassy carbon electrode (5 mm in diameter, 0.196 cm^2^) with deposited electrocatalysts layer and graphite rod, respectively. The reference electrode was a Hg/HgO (1 M NaOH) electrode in alkaline medium. The electrocatalysts layer was prepared by casting 30 μL of slurry onto the glassy carbon electrode pre-polished by 0.05 $\text{μm}$ alumina. The ink slurries (HER: 0.6 mg_cat_ mL^–1^, HOR: 1.0 mg_cat_ mL^–1^) were fabricated by dispersing certain amount of electrocatalysts with water, ethanol and Nafion solution (5 wt%, Dupont) (v_water_ : v_ethanol_ : v_Nafion_ = 1 : 9 : 0.01) under mild sonication for 10 min. The electrocatalyst loading on the RDE was 90 and 150 μg_cat._ cm_disk_^–2^ for HER and HOR, respectively. All potentials in this work were coverted to reversible hydrogen electrode (RHE) according to E(RHE) = E(Hg/HgO) + 0.895 V in 0.1 M KOH or E(RHE) = E(Hg/HgO) + 0.917 V in 1.0 M KOH. The voltage differences between two electrodes were determined by the equilibrium potential of Pt as working electrode in corresponding solutions saturated with H_2_.

For HER, CV curves were recorded in a N_2_-saturated 1.0 M KOH aqueous solution at 25 ^o^C and a scan rate of 20 mV s^–1^ from 0.05 to 0.90 V (vs. RHE). Alkaline HER polarization curves were collected in N_2_-saturated 1.0 M KOH aq. at 25 ^o^C and a rotation speed of 1600 rpm with a negative scan rate of 5 mV s^–1^. The chronopotentiometry tests were utilized to evaluate the electrocatalytic stability of various electrocatalysts.

For HOR, CV curves were recorded in a N_2_-saturated 0.1 M KOH aqueous solution at 25 ^o^C and a scan rate of 50 mV s^–1^ from 0.05 to 0.4 V (vs. RHE). Alkaline HOR polarization curves were collected in H_2_-saturated 0.1 M KOH aq. at 25 ^o^C and a rotation speed of 1600 rpm with a positive scan rate of 10 mV s^–1^ from -0.1 to 0.5 V (vs. RHE). The potential was obtained after the correction of solution resistance, which was measured by electrochemical impedance spectroscopy (EIS) from 200 kHz to 100 mHz with a voltage perturbation of 10 mV at open-circuit voltage.

CO stripping experiments were carried out to estimate the oxophilicity of electrocatalysts. Initially, CO was introduced into 0.1 M KOH aq. for 10 min to achieve a complete monolayer of adsorbed CO on the metal surface with the potential of working electrode maintained at 0.1 V (vs. RHE). Subsequently, N_2_ was introduced for 10 min to completely remove CO from the electrolyte solution, while the potential of working electrode was kept at 0.1 V (vs. RHE). CO stripping was conducted by cycling the potential between 0.05 and 1.0 V (vs. RHE) at a scan rate of 20 mV s^–1^. Background CVs were recorded at the same scan rate immediately after CO stripping procedure was completed.

For HER long-term stability tests, it was assessed by the chronopotentiometric method in 1.0 M KOH at a constant current density of 500 mA cm^–2^ or 1000 mA cm^–2^. The long-term stability of HOR was tested by means of chronoamperometry curves (i ~ t) at an anode potential of 0.4 V (vs. RHE) for 10000 s. For accelerated durability tests, potential cycling was conducted in 1.0 M KOH/0.1 M KOH at a scan rate of 100 mV s^–1^ for 10000/5000 cycles for alkaline HER/HOR. CO stability testing was measured by chronoamperometry test (i ~ t) in 100 ppm CO/H_2_ saturated 0.1 M KOH at 0.1 V for 4000 s.

**SV| DFT Calculations.** The whole DFT calculations were executed utilizing the Vienna Ab-inito Simulation Package (VASP). The exchange-correlation interactions were described by the Perdew-Burke-Ernzerhof (PBE) functional within the generalized gradient approximation (GGA) method. The core-valence effects were implemented by the projected augmented wave (PAW) method. The plane wave basis set was used with 500 eV energy cutoff. The vacuum space was set to 15 Å above the surfaces to avoid periodic interactions. The structural optimization was completed when energy and force convergence were smaller than 1.0×10^–4^ eV and 0.02 eV Å^–1^, respectively.

The Gibbs free energy change (*ΔG*) of each step is calculated using following
equation.

*∆G = ∆E + ∆ZPE - T∆S*  (Equation S2)

where *ΔE* is the electronic energy difference directly obtained from DFT calculations, *ΔZPE* is the zero point energy difference, *T* is the room temperature (298.15 K) and *ΔS* is the entropy change. *ZPE* could be obtained after frequency calculation as follows.

*ZPE =* $\frac{\text{1}}{\text{2}}\text{ }\sum\text{hv}$ (Equation S3)

And the *TS* values of adsorbed species are calculated according to the vibrational frequencies.

*TS*=$\text{ }\text{k}_{\text{B}}\text{T}\text{ [ }\sum_{\text{k}} \text{ln(}\frac{\text{1}}{\text{1-}\text{e}^{\text{-hv/}\text{k}_{\text{B}}\text{T}}}\text{)+ }\sum_{\text{k}} \frac{\text{hv}}{\text{k}_{\text{B}}\text{T}}\text{ }\frac{\text{1}}{{\text{(}\text{e}}^{\text{hv/}\text{k}_{\text{B}}\text{T}}\text{-1)}}\text{+1 ]}$ (Equation S4)

**SVI | Activity Calculations of Alkaline HER.**

The alkaline HER mass activity was calculated by dividing the current density at an overpotential of 50 mV by the mass loading per geometric area.

**SVII | Activity Calculations of Alkaline HOR.**

*j*_k_ of alkaline HOR was extracted by reversible Koutecky-Levich equation as follows.

$\frac{\text{1}}{\text{j}}\text{ = }\frac{\text{1}}{\text{j}_{\text{k}}}\text{+}\frac{\text{1}}{\text{j}_{\text{d}}}$ (Equation S5)

Where *j* is the measured current density, *j*_k_ is kinetic current density, *j*_d_ is diffusion current density defined as

$\text{ η}_{\text{d}}\text{ = -}\frac{\text{RT}}{\text{2}\text{F}}\text{ln}\text{ (1}\text{-}\frac{\text{j}_{\text{d}}}{\text{j}_{\text{l}}}$) (Equation S6)

Where *η*_d_ is the diffusion overpotential, *j*_l_ is the hydrogen mass transport limited current density and can be described by Levich equation, *F* is Faraday’s constant, *R* is the universal gas constant, and *T* is the temperature.

Mass activity (*j*_0,m_) was calcualted by the Bulter-Volmer equation as follows,

$\text{j}_{\text{k}}\text{ = }\text{j}_{\text{0,m}}\text{ (}\text{e }^{\frac{\text{αF}}{\text{RT}}\text{η}}\text{-}\text{e}^{\text{ }\frac{\text{(α-}\text{1}\text{)F}}{\text{RT}}\text{η}}\text{)}$ (Equation S7)

where *α* represents the electron transfer coefficient.

**B. Supplementary Figures**


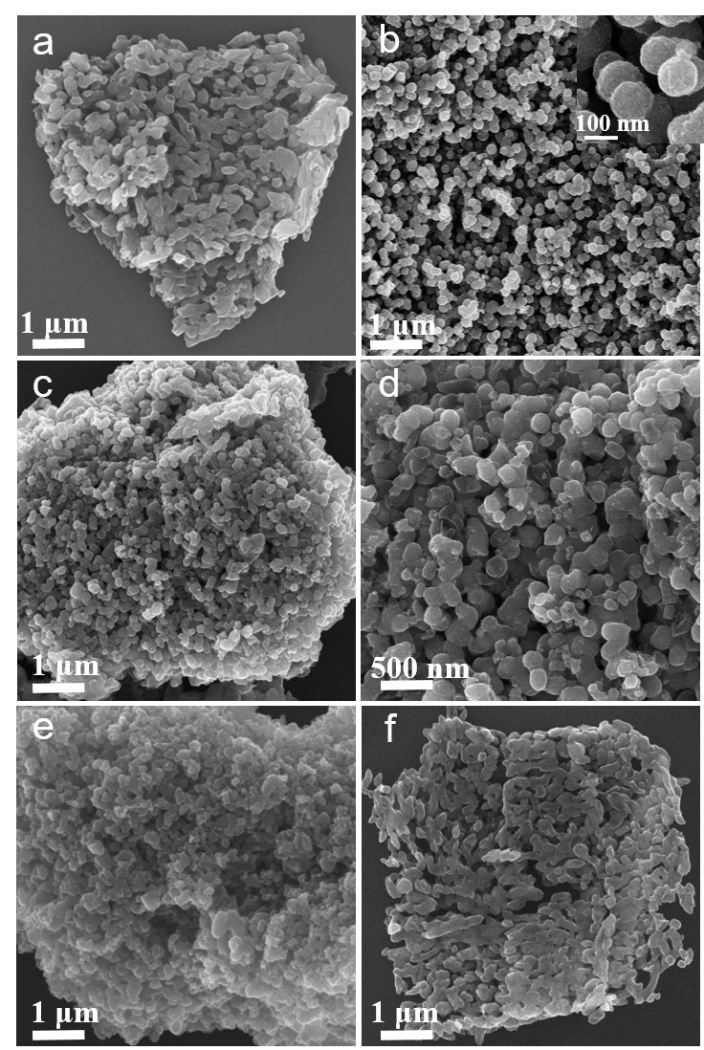


**Figure S1.** SEM images of as-prepared (a) conductive carbon support, (b) TiO_2_ nanospheres (~100 nm), (c and d) F-Ru@TiO_2_, (e) HS-Ru@TiO_2_ and (f) Ru/C samples.


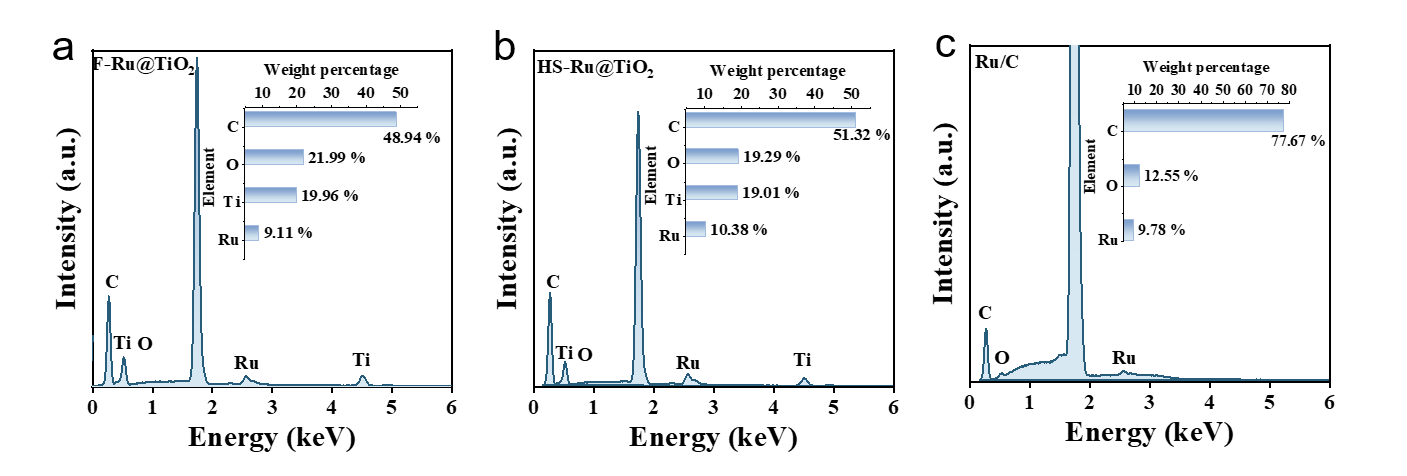


**Figure S2.** EDS analysis of (a) F-Ru@TiO_2_, (b) HS-Ru@TiO_2_ and (c) Ru/C.


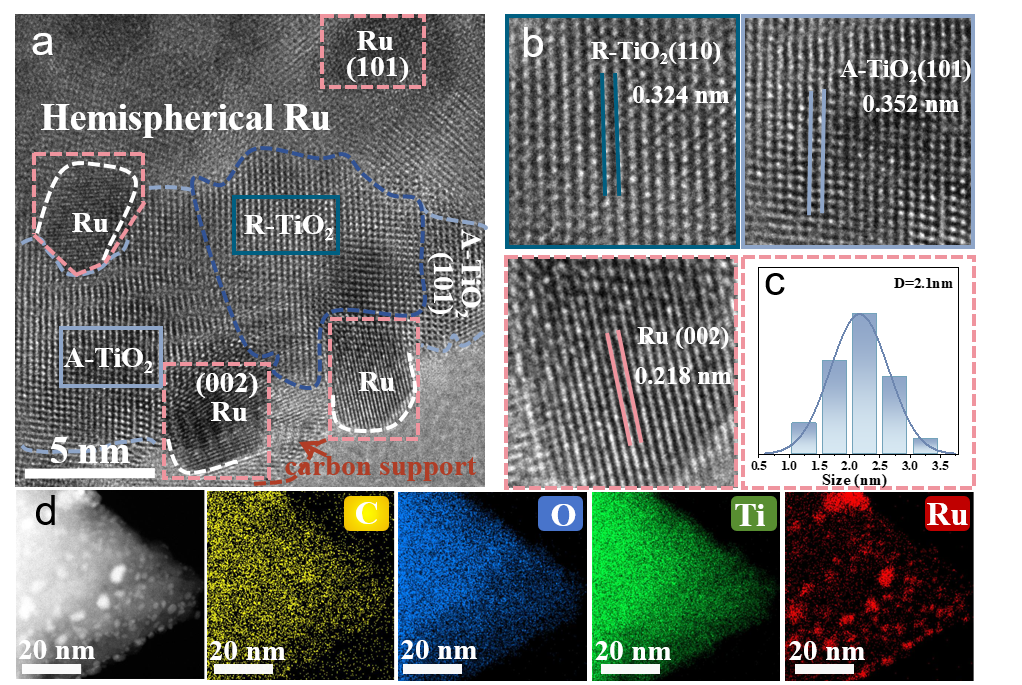


**Figure S3.** (a and b) HRTEM, (c) The particle size distribution of Ru clusters in HS-Ru@TiO_2_, and (d) HAADF with EDS elemental mapping images of HS-Ru@TiO_2_.


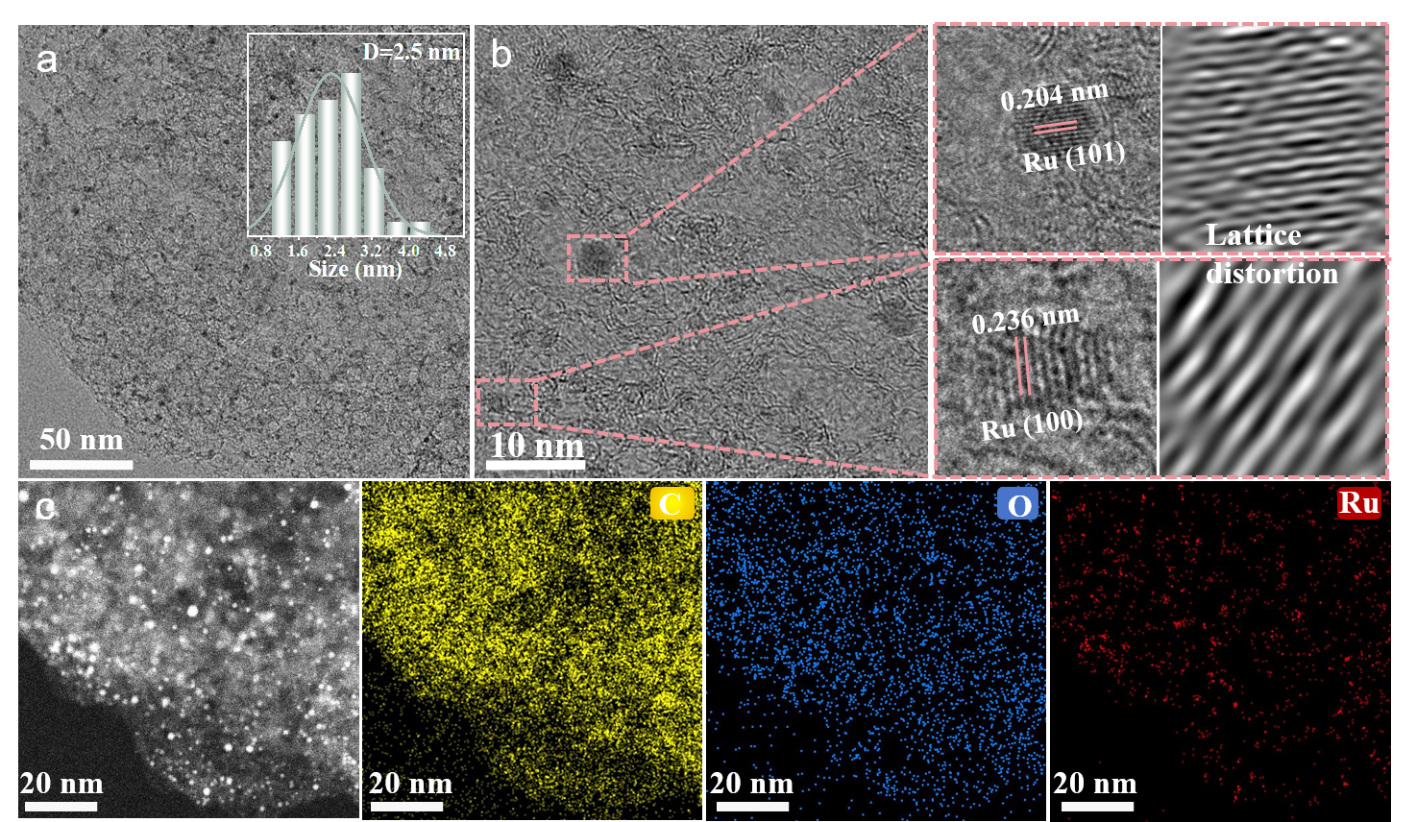


**Figure S4.** (a) TEM, (b) HRTEM and (c) HAADF with EDS elemental mapping images of Ru/C.


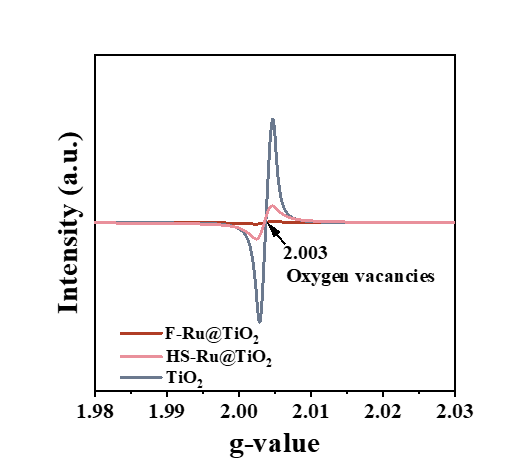


**Figure S5.** The EPR spectra of F-Ru@TiO_2_, HS-Ru@TiO_2_, and original TiO_2_.


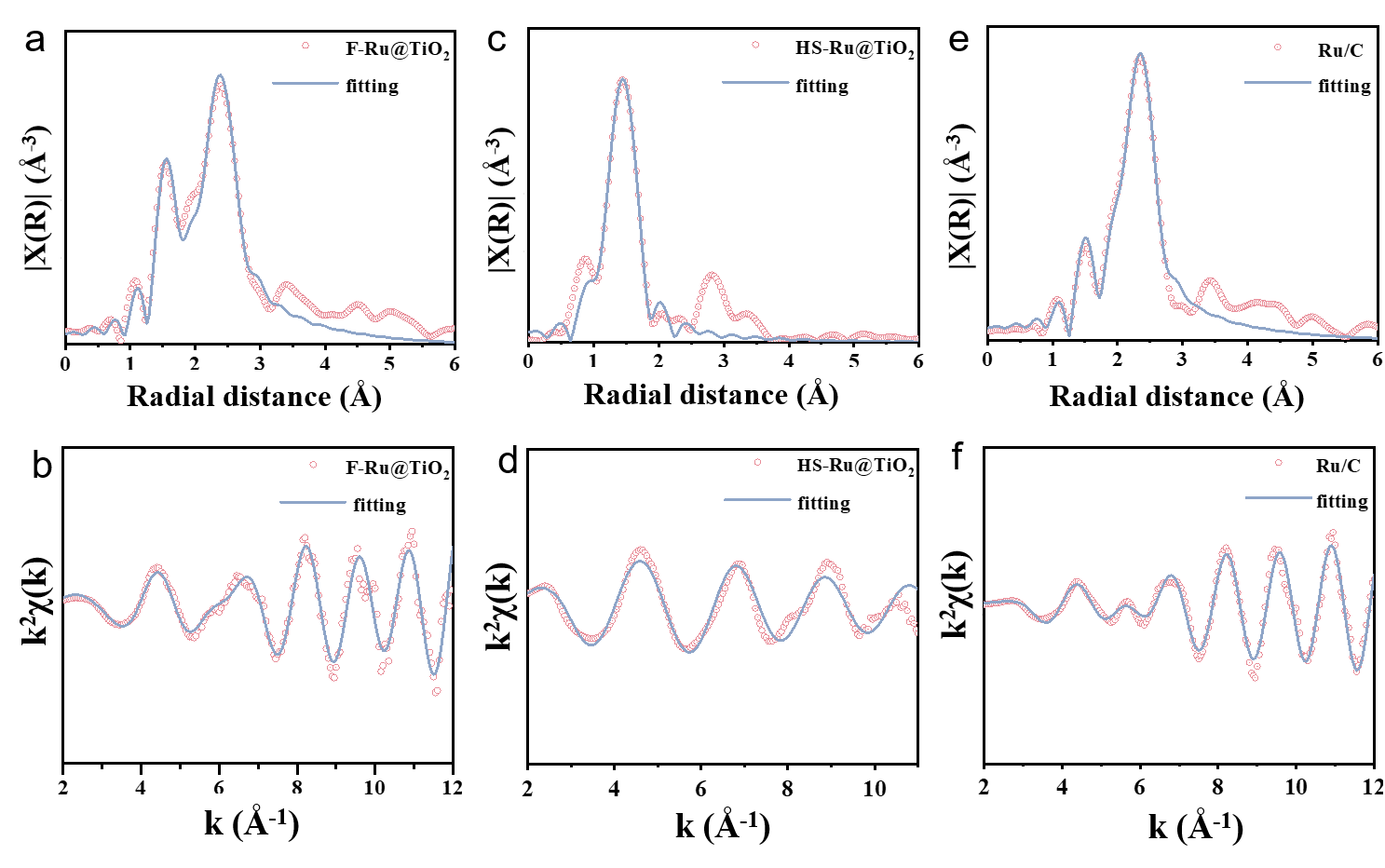


**Figure S6.** Ru K-edge EXAFS experimental and fitting curves of the F-Ru@TiO_2_ at (a) *R* and (b) *k* spaces, HS-Ru@TiO_2_ at (c) *R* and (d) *k* spaces, and Ru/C at (e) *R* and (f) *k* spaces.


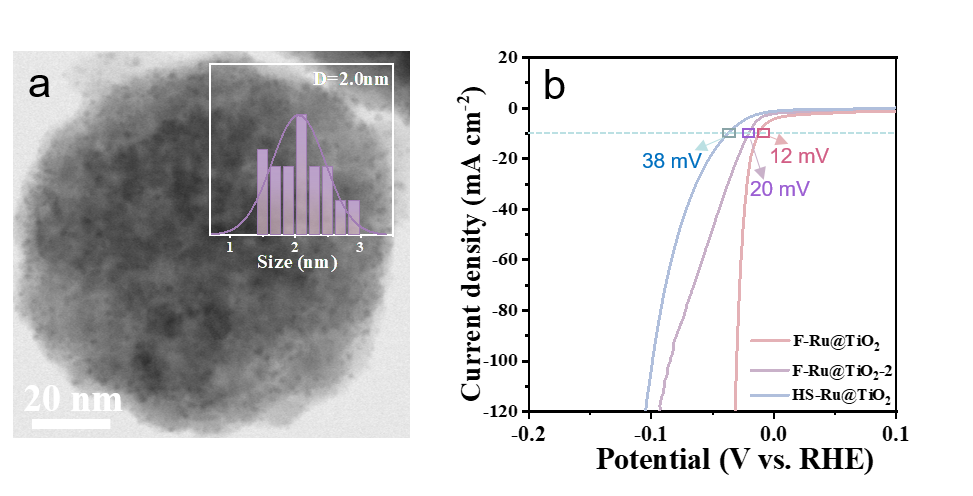


**Figure S7.** (a) The TEM image of F-Ru@TiO_2_-2, prepared by increasing the impregnated amount of RuCl_3_ to 40 mg, having a size comparable to those found in HS-Ru@TiO_2_ (~2 nm). (b) HER polarization curves of F-Ru@TiO_2_, F-Ru@TiO_2_-2, HS-Ru@TiO_2_.

F-Ru@TiO_2_-2, which has Ru particle sizes similar to those in HS-Ru@TiO_2_, exhibits higher HER performance relative to HS-Ru@TiO_2_ (Figure S7b), further confirming the significance of the coexistence of Ru-Ti and Ru-O-Ti interfacial bonds. Additionally, it is evident that as the Ru particle size increases from 1 nm to 2 nm within the F-Ru@TiO_2_, there is a corresponding decrease in HER activity. This observation highlights the critical influence of interfacial chemical bonds and particle size on the electrocatalytic efficiency of these materials, but the former plays a prominent role.


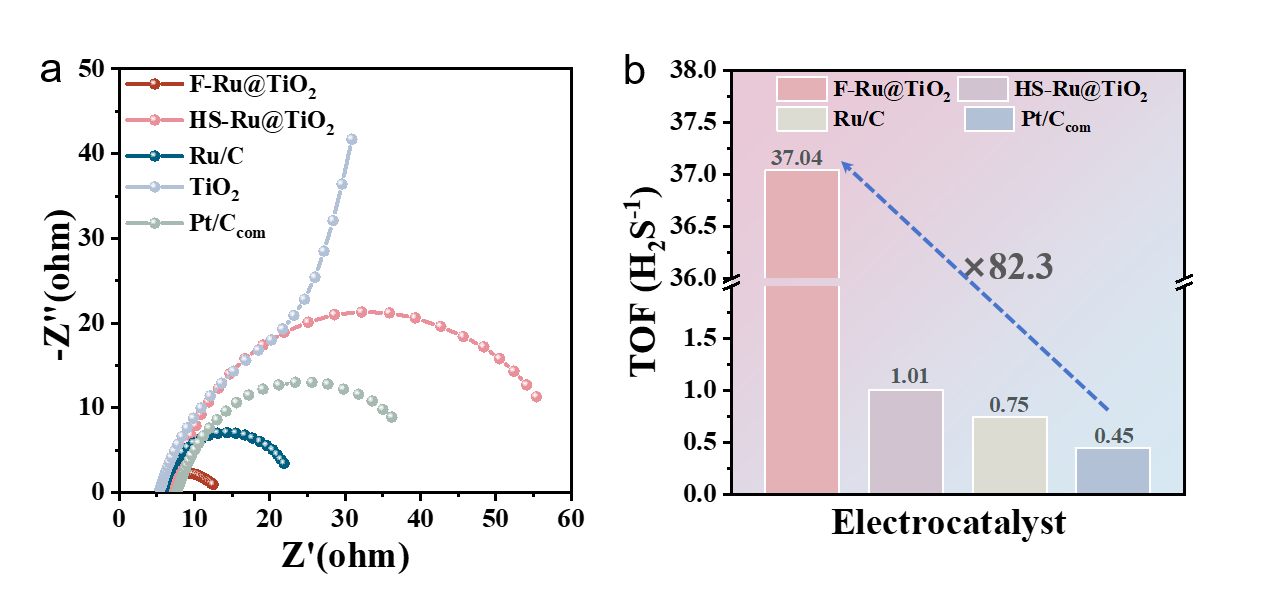


**Figure S8.** (a) Nyquist plots at 5 mV overpotential and (b) TOF values at 50 mV overpotential of F-Ru@TiO_2_, HS-Ru@TiO_2_, Ru/C and Pt/C during HER process under 1.0 M KOH solution.


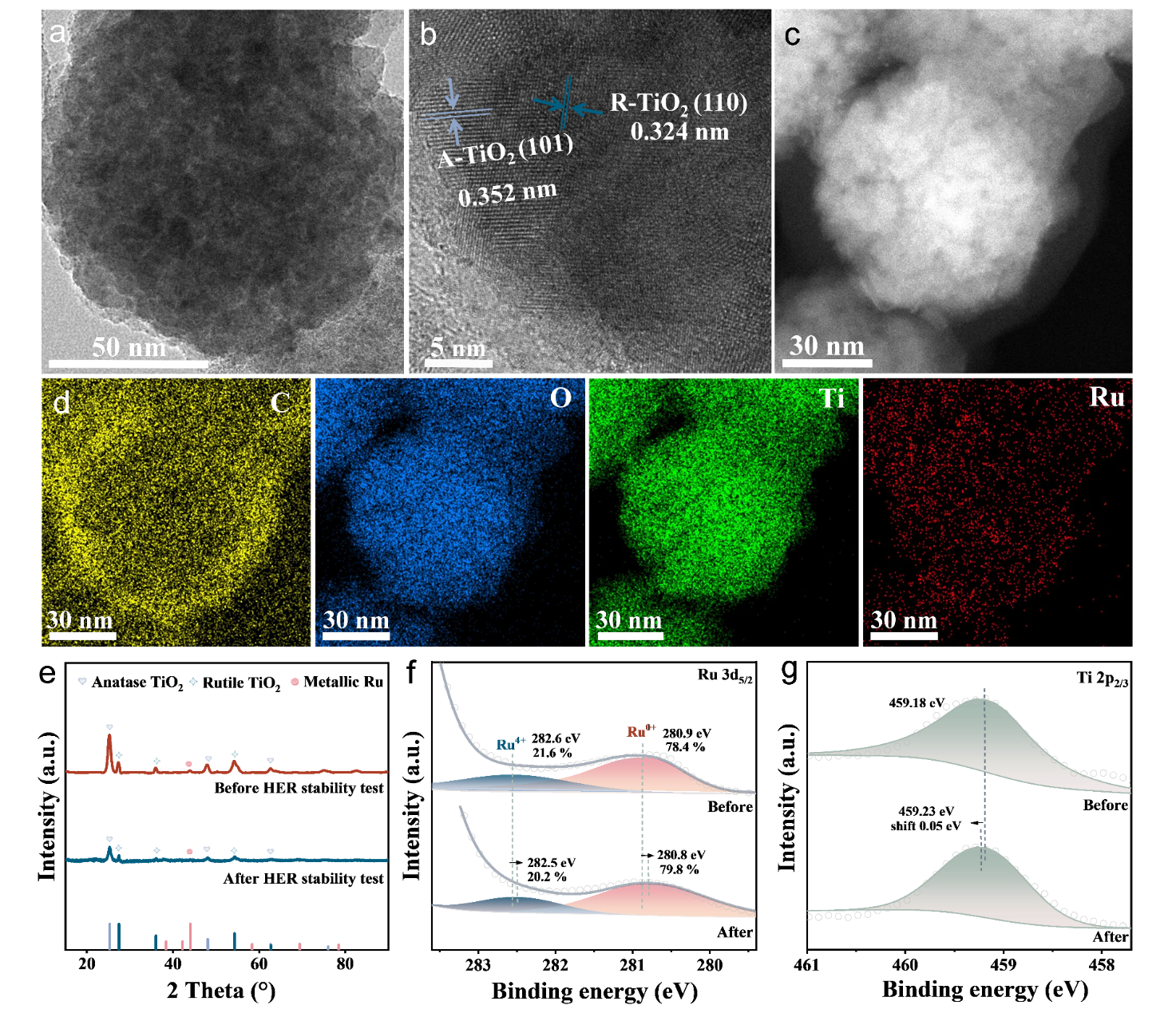


**Figure S9.** (a) TEM image, (b) HRTEM image, (c) HAADF-STEM image and (d) the corresponding elemental mapping of the F-Ru@TiO_2_ after the stability test for HER. (e) XRD patterns, (f) Ru 3d XPS spectra and (g) Ti 2p XPS spectra of the F-Ru@TiO_2_ after the stability test for HER.


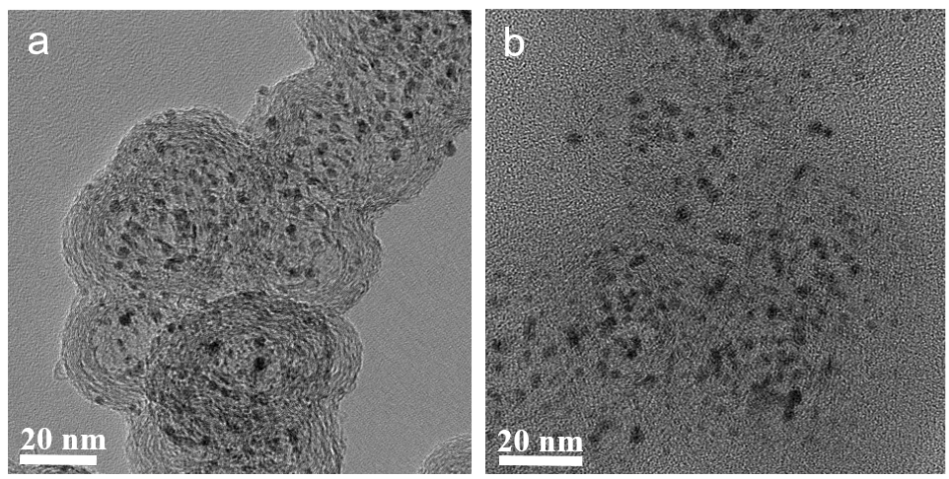


**Figure S10.** (a) TEM image of commercial Pt/C before the stability test for HER. (b) TEM image of commercial Pt/C after the stability test for HER.


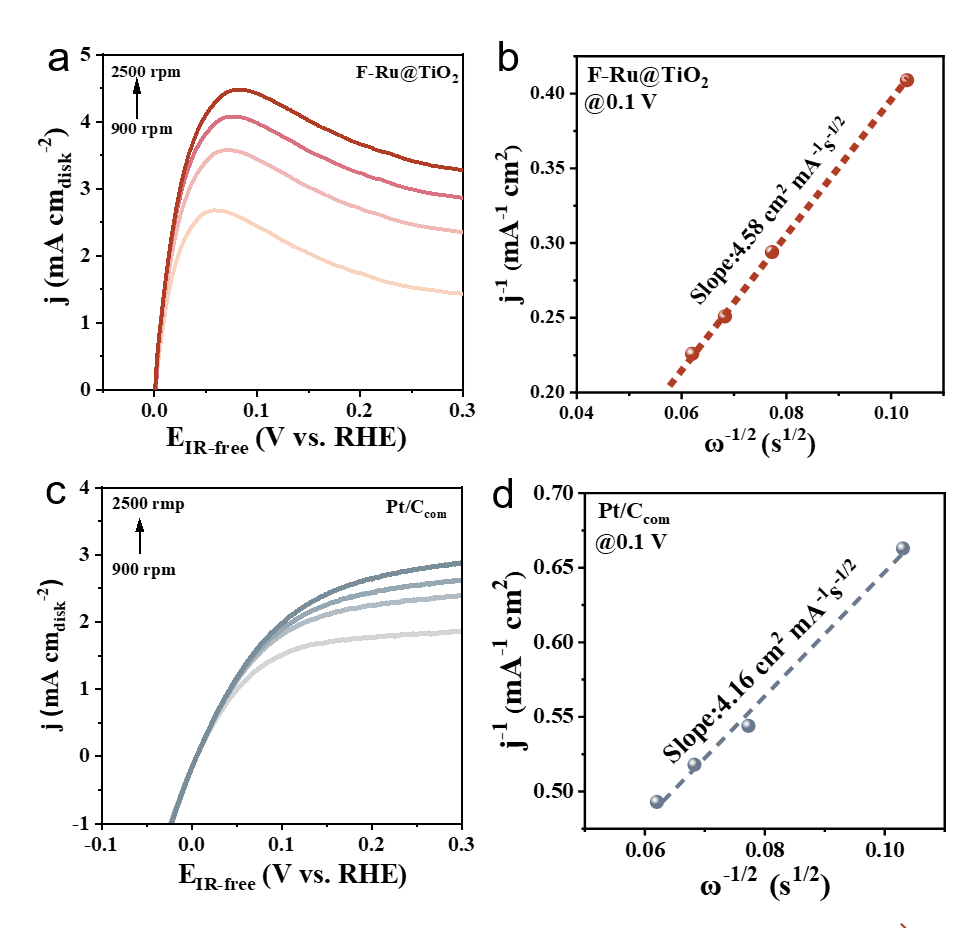


**Figure S11.** (a) HOR polarization curves of the (a) F-Ru@TiO_2_ and (c) Pt/C_com_ electrocatalysts measured at various rotational speeds. The corresponding Koutecky-Levich plots obtained at 0.1 V overpotential of the (b) F-Ru@TiO_2_ and (d) Pt/C_com_ electrocatalysts.


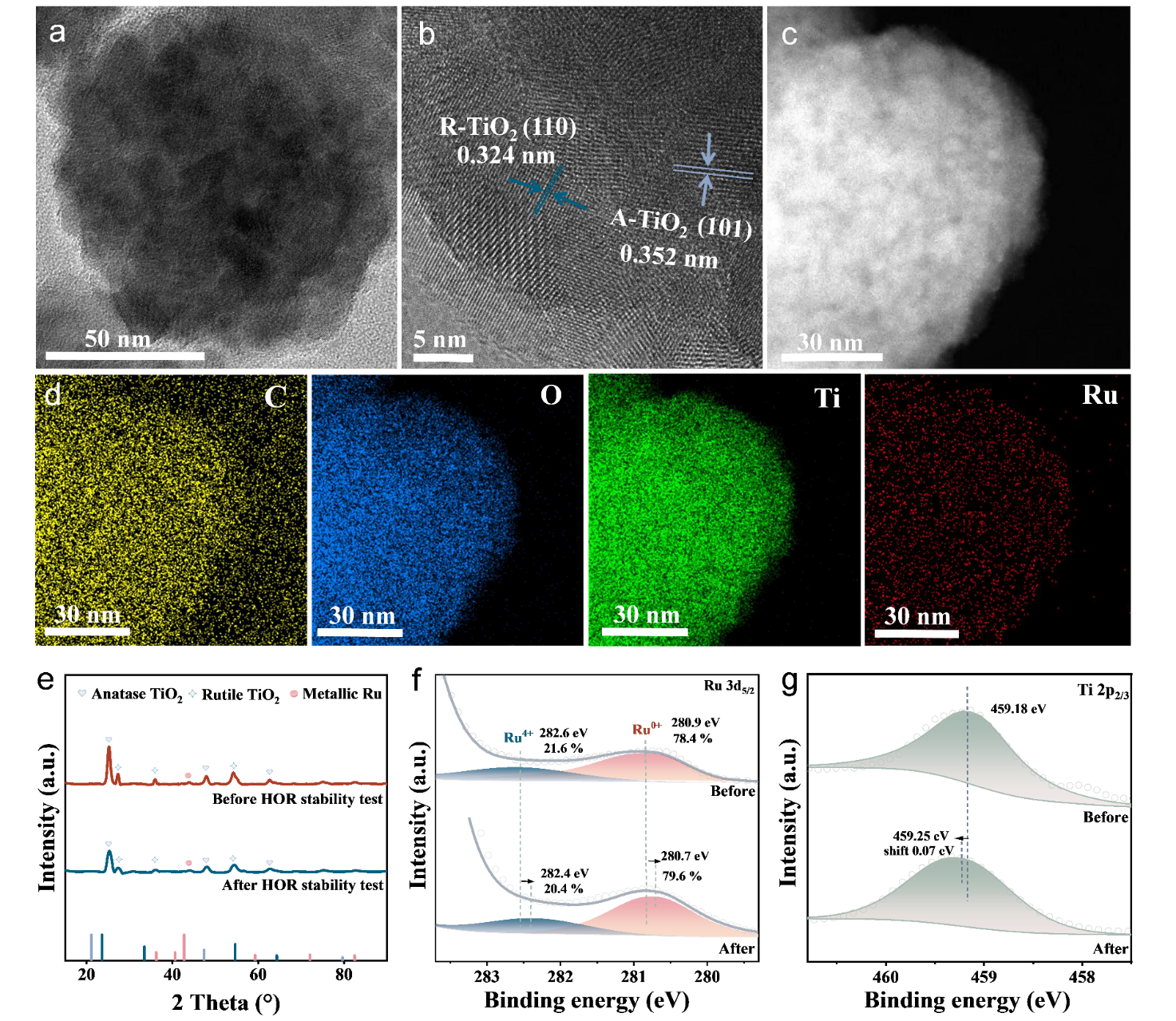


**Figure S12.** (a) TEM image, (b) HRTEM image, (c) HAADF-STEM image and (d) the corresponding elemental mapping of the F-Ru@TiO_2_ after the stability test for HOR. (e) XRD patterns, (f) Ru 3d XPS spectra and (g) Ti 2p XPS spectra of the F-Ru@TiO_2_ after the stability test for HOR.


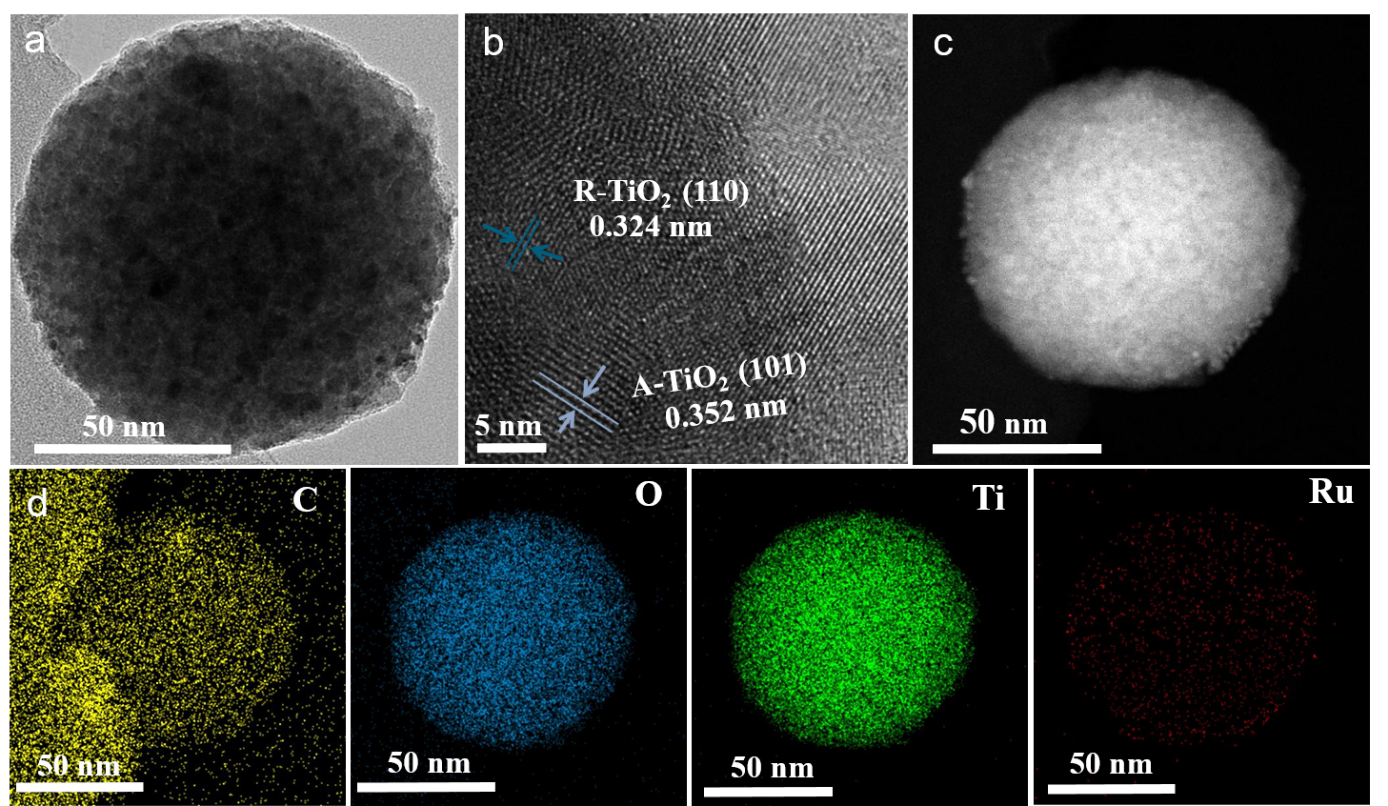


**Figure S13.** (a) TEM image, (b) HRTEM image, (c) HAADF-STEM image and (d) the corresponding elemental mapping of the F-Ru@TiO_2_ after the CO/H_2_ chronoamperometry test.


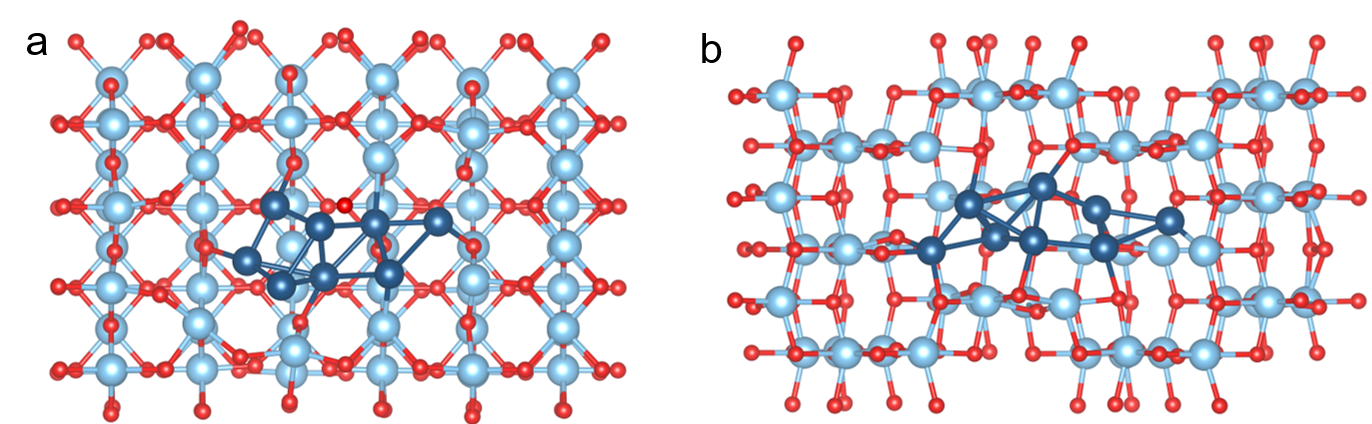


**Figure S14.** (a) F-Ru@Rutile-TiO_2_ and F-Ru@Anatase-TiO_2_ models from top view for DFT calculations.


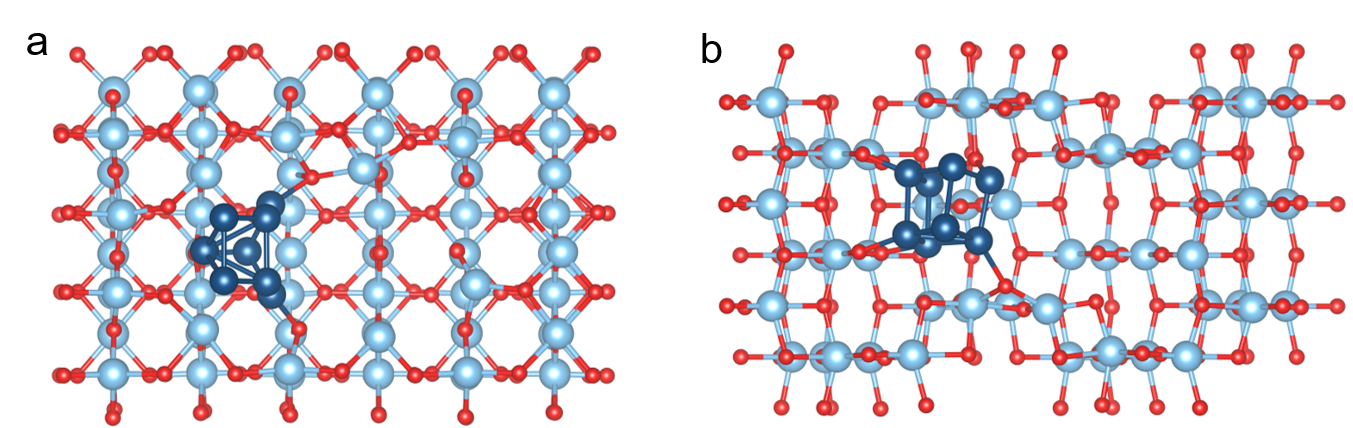


**Figure S15.** (a) HS-Ru@Rutile-TiO_2_ and (b) HS-Ru@Anatase-TiO_2_ models from top view for DFT calculations.


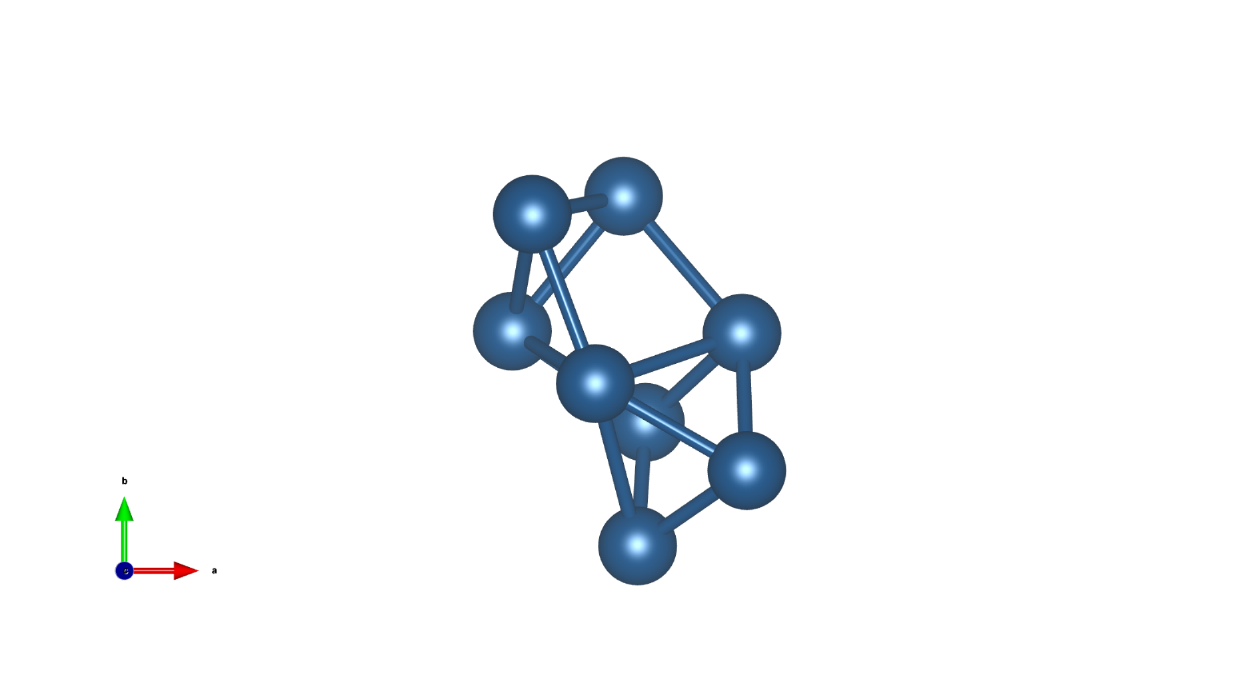


**Figure S16.** Ru_NC_ model from top view for DFT calculation.


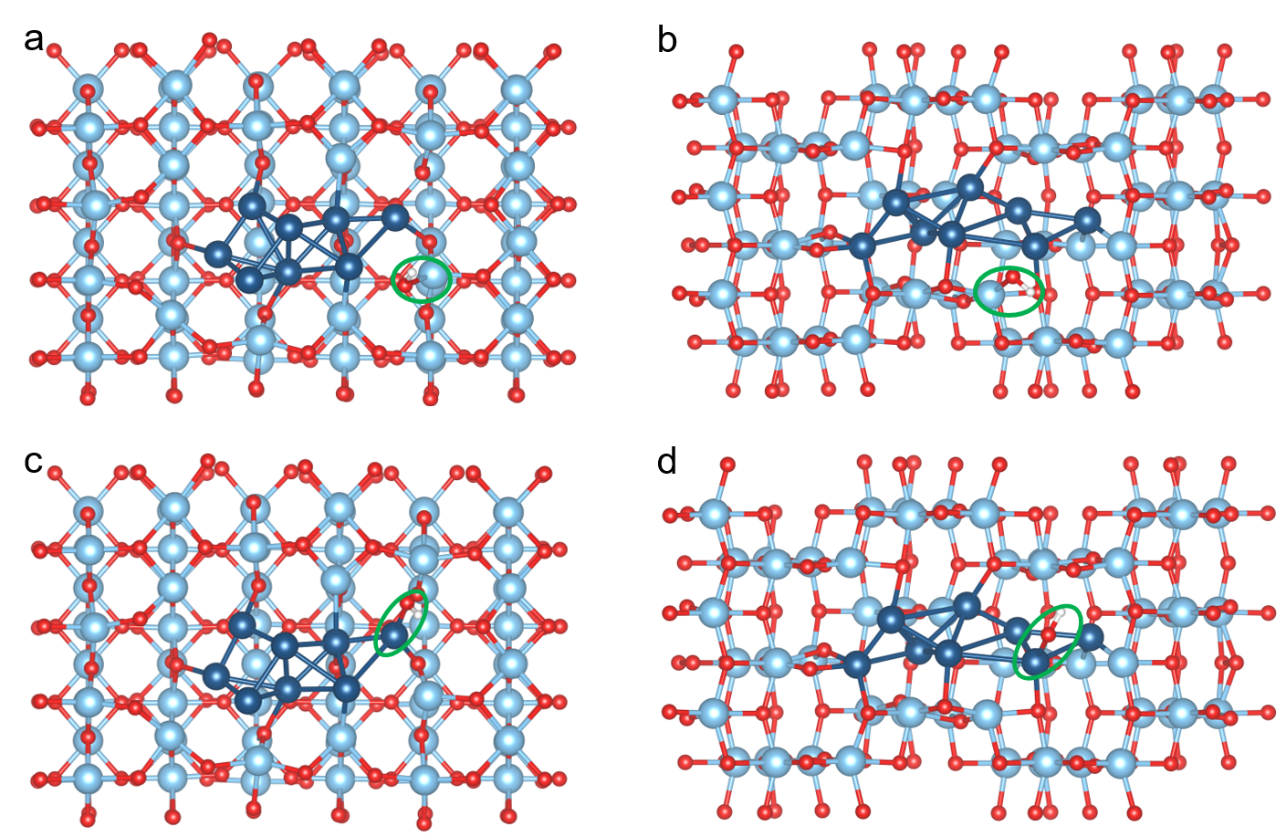


**Figure S17.** The theoretical structures of (a) F-Ru@Rutile-TiO_2_ and (b) F-Ru@Anatase-TiO_2_ after the optimal adsorption of OH on the Ti site of Ru-O-Ti interfacial chemical bond. The theoretical structures of (c) F-Ru@Rutile-TiO_2_ and (d) F-Ru@Anatase-TiO_2_ after the optimal adsorption of OH on the Ru site of Ru-O-Ti interfacial chemical bond. The red, dark blue, light blue and white balls represent O, Ru, Ti and H atoms, respectively.

**
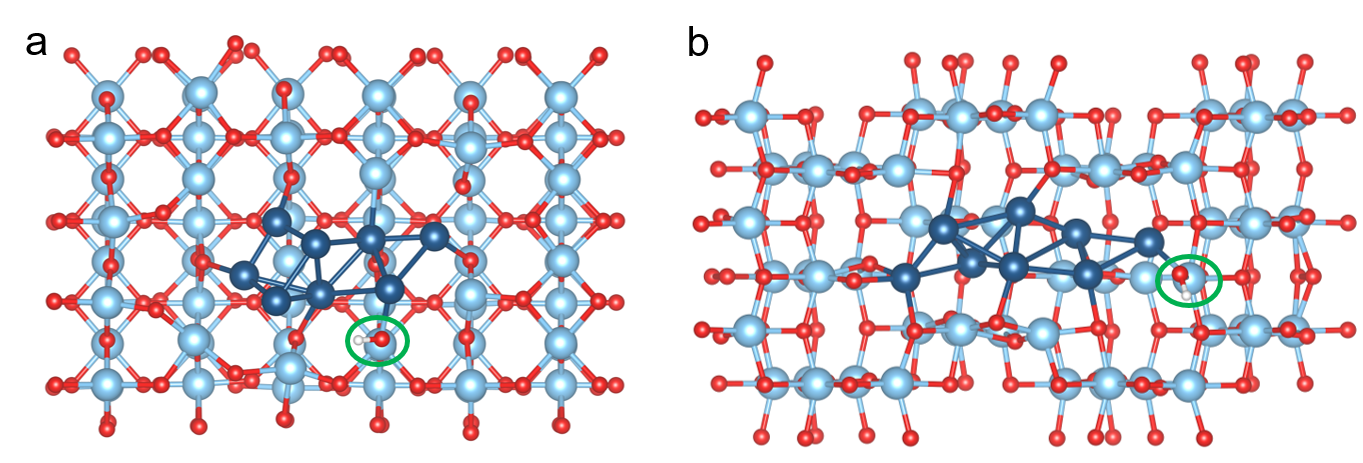
**

**Figure S18.** The theoretical structures of (a) F-Ru@Rutile-TiO_2_ and (b) F-Ru@Anatase-TiO_2_ after the optimal adsorption of OH on the Ti site of Ru-Ti interfacial chemical bond. The red, dark blue, light blue and light pink balls represent O, Ru, Ti and H atoms, respectively.

**
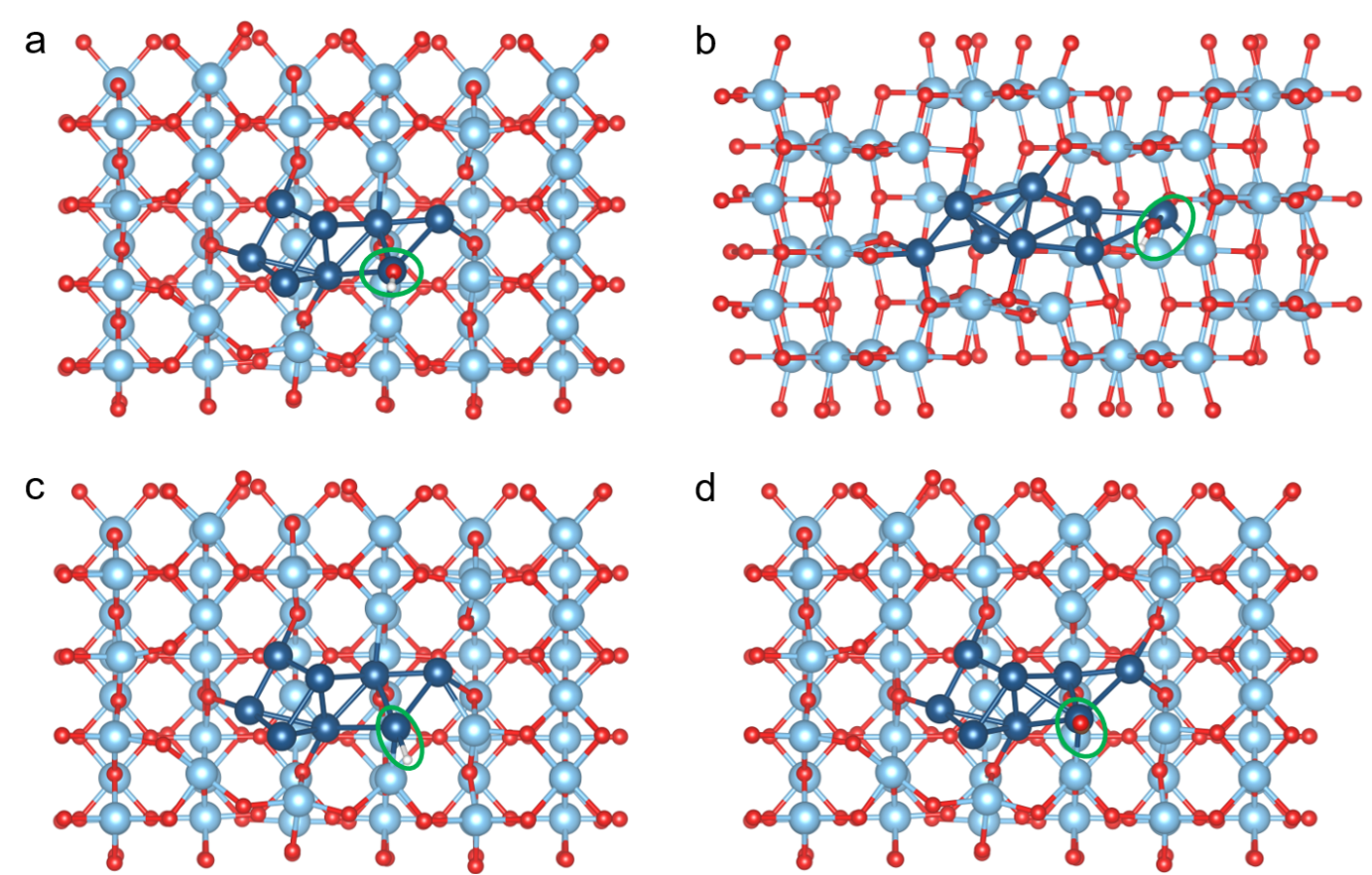
**

**Figure S19.** The theoretical structures of (a) F-Ru@Rutile-TiO_2_ and (b) F-Ru@Anatase-TiO_2_ after the optimal adsorption of OH on the Ru site of Ru-Ti interfacial chemical bond. The theoretical structures of F-Ru@Rutile-TiO_2_ after the optimal adsorption of (c) H and (d) CO on the Ru site of Ru-Ti interfacial chemical bond. The brown, red, dark blue, light blue and light pink balls represent C, O, Ru, Ti and H atoms, respectively.

**
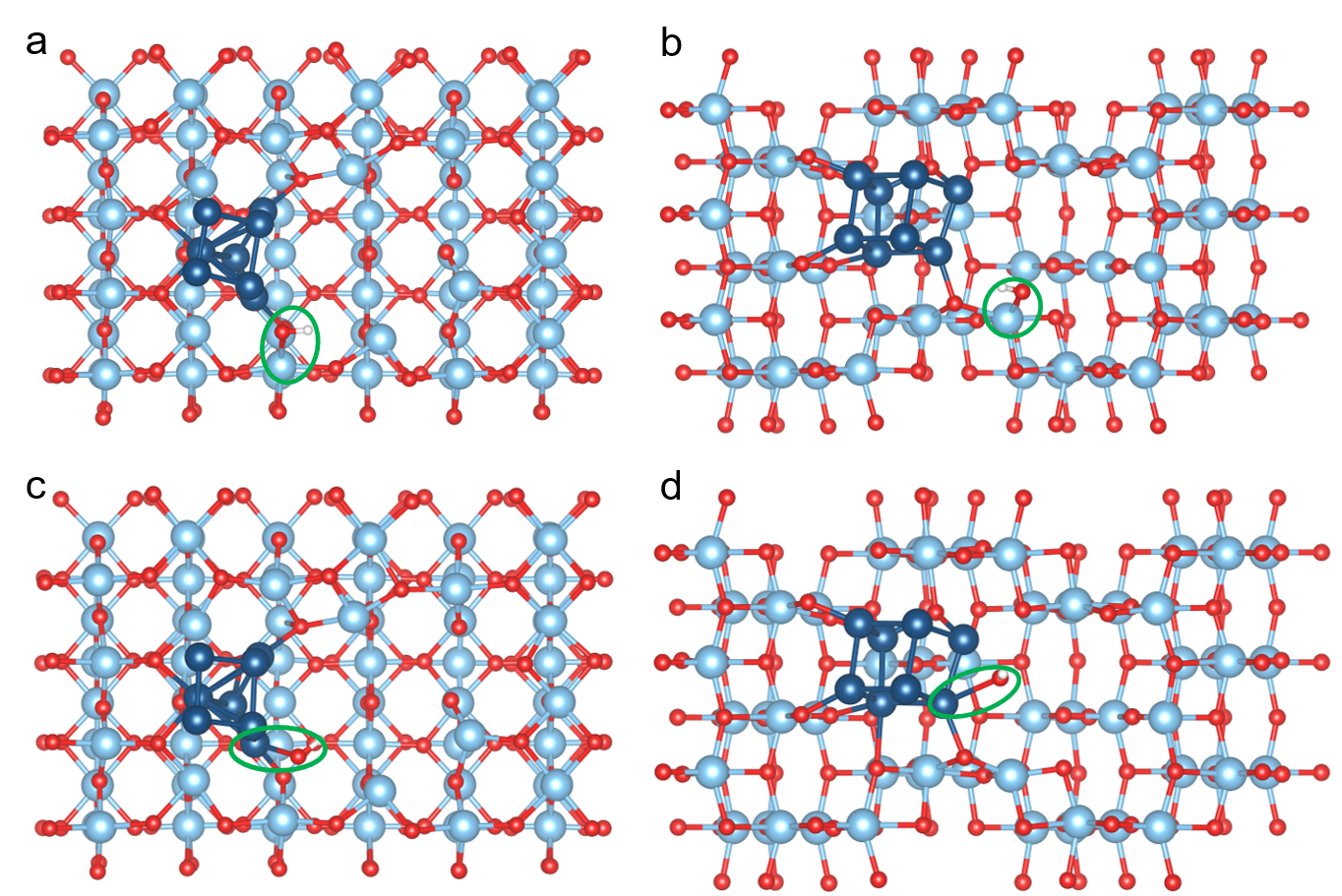
**

**Figure S20.** The theoretical structures of (a) HS-Ru@Rutile-TiO_2_ and (b) HS-Ru@Anatase-TiO_2_ after the optimal adsorption of OH on the Ti site of Ru-O-Ti interfacial chemical bond. The theoretical structures of (c) HS-Ru@Rutile-TiO_2_ and (d) HS-Ru@Anatase-TiO_2_ after the optimal adsorption of OH on the Ru site of Ru-O-Ti interfacial chemical bond. The red, light blue, dark blue, and white balls represent O, Ti, Ru and H atoms, respectively.


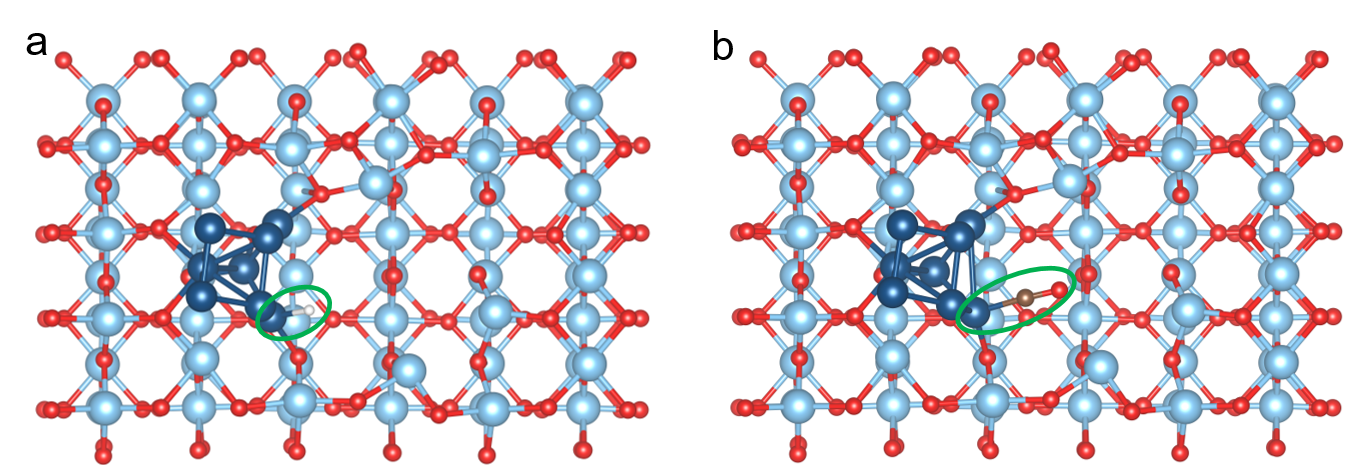


**Figure S21.** The theoretical structures of HS-Ru@Rutile-TiO_2_ after the optimal adsorption of (a) H and (b) CO on the Ru site of Ru-O-Ti interfacial chemical bond. The brown, red, light blue, dark blue, and white balls represent C, O, Ti, Ru and H atoms, respectively.


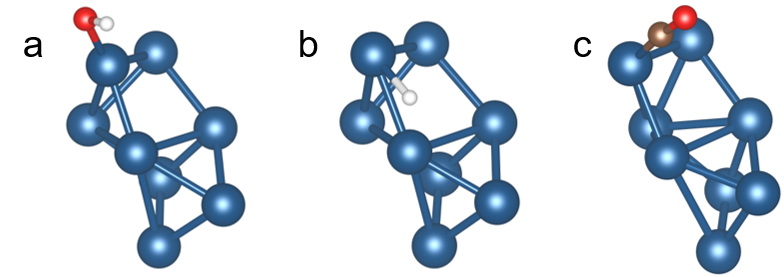


**Figure S22.** The theoretical structures of Ru_NC_ after the optimal adsorption of (a) OH, (b) H and (c) CO. The brown, red, dark blue, and white balls represent C, O, Ru and H atoms, respectively.

**C. Supplementary Tables**

**Table S1.** Fitting results and parameters for the EXAFS of the F-Ru@TiO_2_, HS-Ru@TiO_2_, Ru/C, Ru foil, and RuO_2_.

| **Sample** | **Shell** | ***CN*^a^** | ***R* (Å)^b^** | ***σ*^2^ (Å^2^)^c^** | ***ΔE_0_*** **(eV)^d^** | ***R* factor (%)^e^** |
| --- | --- | --- | --- | --- | --- | --- |
| Ru foil | Ru-Ru | 12 | 2.68 | 0.0039 | -5.2±0.7 | 0.0035 |
| RuO_2_ | Ru-O | 6.0±0.4 | 1.97 | 0.0018 | -0.8±0.5 | 0.0048 |
|  | Ru-Ru | 9.6±2.0 | 3.15 | 0.0097 | -2.9±0.7 |  |
|  | Ru-Ru | 4.8±1.4 | 3.59 | 0.0012 | 5.8±0.7 |  |
| F-Ru@TiO_2_ | Ru-O | 2.9±0.3 | 2.10 | 0.0032 | 5.4±0.5 | 0.0096 |
|  | Ru-Ru(Ti) | 1.4±0.3 | 2.70 | 0.0003 | -1.0±0.8 |  |
| HS-Ru@TiO_2_ | Ru-O | 5.5±0.3 | 1.98 | 0.0049 | -1.5±0.3 | 0.0021 |
| Ru/C | Ru-O | 2.4±0.4 | 2.09 | 0.0056 | 4.6±1.0 | 0.0091 |
|  | Ru-Ru | 3.2±0.5 | 2.68 | 0.0022 | -5.9±0.6 |  |

*^a^CN*: coordination numbers; *^b^R*: bond distance; *^c^σ*^2^: Debye-Waller factors; *^d^*Δ*E*_0_: the inner potential correction; *^e^R* factor: goodness of fit.

**Table S2.** Comparison of HER activity measured for F-Ru@TiO_2_ with other reported HER electrocatalysts in 1 M KOH electrolyte.

| **Catalyst** | **η_10_ (mV)** | **η_500_ (mV)** | **η_1000_ (mV)** | **Tafel Slope**  **(mV dec^-1^)** | **Reference** |
| --- | --- | --- | --- | --- | --- |
| F-Ru@TiO_2_ | 12 | 45 | 74 | 25.2 | This work |
| MoO_2_@Ru NT | 22 | 89 | 131 | 29.1 | ^1^ |
| Ru-Mo_2_C@CNT | 15 | 56 | 78 | 26 | ^2^ |
| Ru_x_SACs@FeCo-LDH | / | 84 | 117 | 53 | ^3^ |
| Ru_SA/NP_-PNCFs | 24 | 75 | 132 | 21.7 | ^4^ |
| Ni-MoN | 24 | 104 | 136 | 35.5 | ^5^ |
| RFNOH-10 | 13 | 103 | 152 | 30 | ^6^ |
| Pt/8-NCNT | 17 | 107 | 153 | 33.3 | ^7^ |
| Ni_3_Sn_2_-NiSnO_x-2_ | 14 | 111 | 165 | 68.8 | ^8^ |
| FeIr/NF | 31.1 | 125 | 204 | 66.95 | ^9^ |
| Cu-m/Cu-W/NiCo-LDH | 15 | 139 | 190 | 50.5 | ^10^ |
| NiMoO_x_/NiMoS | 38 | 174 | 236 |  | ^11^ |
| NMFSOH | 54 | 175 | 200 | 41 | ^12^ |
| Ru-1.0 | 19 | 119 | 196 | 25.3 | ^13^ |
| R-CoC_2_O_4_@MXene | 28 | 157 | 216 | 43 | ^14^ |
| Ni_3_S_2_/Cr_2_S_3_@NF | / | 207 | 227 | 87 | ^15^ |
| Ru_NP_-Ru_SA_@CFN-800 | 33 | 154 | 251 | 37.16 | ^16^ |
| Ru_6_/SRO | 18 | 154 | 251 | 22 | ^17^ |
| Cu-FeOOH/Fe_3_O_4_ | / | 285 | 349 | 11 | ^18^ |
| Pd_4_S/Pd_3_P_0.95_ | 42 | 387 | 486 | 27.8 | ^19^ |

**Table S3.** Reported alkaline HER mass activity and stability testing of PGM-based electrocatalysts.

| **Catalyst** | **Mass Activity**  **(A g_mental_^–1^)** | **Stability**  **(Cycling)** | **Reference** |
| --- | --- | --- | --- |
| F-Ru@TiO_2_ | 70.69@50 mV | 10000 | This work |
|  |  | 100 h@500 mA cm^-2^ |  |
|  |  | 100 h@1000 mA cm^-2^ |  |
| PBN-300-Ir | 51.6@70 mV | 38 h@10 mA cm^-2^ | ^20^ |
| Ru_1_/D-NiFe LDH | 14.65@100 mV | 2000  50 h@100 mA cm^-2^ | ^21^ |
| Ru_1_-Mo_2_C | 8.67@100 mV | 10000  140 h@56.8 mV | ^22^ |
| Pt-AC/Cr-N-C | 7.90@50 mV | 24 h@10 mA cm^-2^ | ^23^ |
| RuIr@NrC | 6.97@100 mV | 2000 | ^24^ |
| Pt/CoNC | 6.90@50 mV | 5000  100 h@10 mA cm^-2^ | ^25^ |
| Pt-Ni ASs | 5@50 mV | 10000 | ^26^ |
| Pt/Ni_3_S_2_/NF | 5.52@150 mV | 20 h@20 mA cm^-2^ | ^27^ |
| Ru_1,n_-NC | 4.50@50 mV | 5000  45 h@15 mV | ^28^ |
| Pt-Ni NTAs | 4.27@50 mV | 48 h@10 mA cm^-2^  48 h@20 mA cm^-2^  48 h@50 mA cm^-2^ | ^29^ |
| Ru-Cu-0.2 | 3.96@50 mV | 70 h@10 mA cm^-2^ | ^30^ |
| Ru_x_-V_8_C_7_/C | 2.29@40 mV | 45 h@10 mA cm^-2^ | ^31^ |
| 2D-PtND/LDH | 2.15@50 mV | 25 h@100 mA cm^-2^ | ^32^ |
| Pt_1_/NMHCS | 2.07@50 mV | 3000 | ^33^ |
| Pt-AC/HMCS | 1.90@50 mV | 3000  20000 s@10 mA cm^-2^ | ^34^ |
| Rh/NiFeRh-LDH | 1.61@50 mV | 1000  20000 s@100 mV | ^35^ |
| Ru_0.3_SrTi_0.7_O_3-δ_ | 1.50@50 mV | 100  200 h@10 mA cm^-2^ | ^36^ |

**Table S4.** Comparison of HOR activity measured for F-Ru@TiO_2_ with other reported HOR electrocatalysts in 0.1 M KOH electrolyte.

| **Catalyst** | **Rotating Rate (rpm)** | ***j*_0,m_**  **(A g_PGM_^-1^)** | **Stability**  **(Cycling)** | \| **Continuous**  **Potential** \| \| --- \|   **(V vs. RHE)** | **Reference** |
| --- | --- | --- | --- | --- | --- | --- |
| F-Ru@TiO_2_ | 1600 | 3155 | 5000 | 0.5 | This Work |
|  |  |  | 3h (0.4 V, i-t) |  |  |
| i-ZnIn-PR/C | 1600 | / | 2000  3h (0.1 V, i-t) | 0.35 | ^37^ |
| Pd_SA_/N,S-OPC | 1600 | 3365 | 3000  45h (0.1 V, i-t) | 0.1 | ^38^ |
| N-Ru/C | 1600 | 540 | 1000 | 0.15 | ^39^ |
| fcc-Ru/C | 1600 | 120 | 5000 | 0.2 | ^40^ |
| Ru_SA_-O_4_/Ru_NP_ | 1600 | / | 1000 | 0.2 | ^41^ |
| MoO*_x_*-Ru fcc | 1600 | 1660 | 20000 s  (i-t) | 0.1 | ^42^ |
| IO-Ru-TiO_2_/C | 1600 | 87.3 | 3600 s  (0.6 V, i-t) | 0.9 | ^43^ |
| Mn_1_O*_x_*(OH)*_y_*@Ru/C | 1600 | ~71 | 3 h  (0.7 V, i-t) | 1.0 | ^44^ |
| Ru-Cr_1_(OH)_x_ | 1600 | 96.6 | 50 h (i-t) | 0.15 | ^45^ |
| Ru-WC_x_ | 1600 | 471 | 2000  40 h (i-t) | 0.3 | ^46^ |
| Ru-TiO/TiO_2_@NC | 2500 | 42 | 3 h  (i-t) | 0.2 | ^47^ |
| Ru/RuO_2_ SNSs/C | 1600 | 487 | 10000s  (i-t) | 0.2 | ^48^ |
| Ru/RuO_2_/C in-plane heterostructures | 1600 | 443 | 8 h  (i-t) | 0.3 | ^49^ |
| Ru/Ni-NiO@C | 1600 | 44.4 | 1000 | 0.2 | ^50^ |
| Mo-Ru-2/C | 1600 | 437.5 | 1000 | 0.2 | ^51^ |
| Ru-Ru_2_P/C | 1600 | 375 | 1000 | 0.2 | ^52^ |
| Ru_2_P/C | 1600 | 270 | 1000 | 0.1 | ^53^ |
| Ru_2.3_Ni_1_/C | 1600 | 38.8 | 3000 | 0.3 | ^54^ |
| RuFe_0.1_ NS/C | 1600 | 234 | 1000 | 0.07 | ^55^ |
| *di*-RuNi MLNS/C | 1600 | ~153 | 3 h  (i-t) | 0.15 | ^56^ |
| Pb_1.04_-Ru_92_Cu_8_/C | 1600 | 44.7 | 1 h  (i-t) | 0.15 | ^57^ |
| Ru@C-280 | 1600 | 343.5 | 2000 | 0.2 | ^58^ |
| Ru/Cu-Cu_2_O@C | 1600 | ~263 | 1000 | 0.15 | ^59^ |
| Ru-SO_4_ | 1600 | 426 | 1000 | 0.1 | ^60^ |
| Ru/PEI-XC | 2500 | 134.6 | 1000 | 0.2 | ^61^ |
| Ru/NC@WOC | 1600 | 74.7 | 1000 | 0.15 | ^62^ |
| Ru_c_/NHCS | 1600 | 725 | 5000 | 0.2 | ^63^ |
| Ru_3_Sn_7_/C | 1600 | 291 | 1000 | 0.15 | ^64^ |
| Ru-WO_3_-20% | 1600 | ~1300 | 10000s  (i-t) | 0.12 | ^65^ |
| V-S-Ru/C | 1600 | 340.18 | 1000 | 0.6 | ^66^ |

**Table S5.** Comparison of CO tolerance between F-Ru@TiO_2_ and other reported catalysts.

| **Catalyst** | **CO Tolerance** | **Time**  **(s)** | **CO**  **Concentration** | **Reference** |
| --- | --- | --- | --- | --- |
| F-Ru@TiO_2_ | 16.0% | 4000 | 100 ppm | This Work |
| Ru/Ti_4_O_7_ | 21.45% | 3600 | 10 ppm | ^67^ |
| Ru@NC/C-400 | 9.5% | 1800 | 100 ppm | ^68^ |
| Pb_1.04_-Ru_92_Cu_8_/C | 12.8% | 3600 | 100 ppm | ^57^ |
| PdNiRuIrRh/C | 19% | 3600 | 100 ppm | ^69^ |
| *di*-RuNi MLNS/C | 47.7% | 3600 | 100 ppm | ^56^ |
| Ru/RuO_2_ SNSs/C | 21.6% | 3000 | 100 ppm | ^48^ |
| RuNi/NC | 10% | 1800 | 200 ppm | ^70^ |
| fcc_0.42_Ru-Sn/C | 10% | 2000 | 1000 ppm | ^71^ |
| Ru@TiO_2_ | 12.4% | 2000 | 1000 ppm | ^72^ |
| Ru/Ru_x_Fe_3−x_O_4_ | 20.3 | 500 | 1000 ppm | ^73^ |
| Pt_6_ NCs | 20% | 600 | 1000 ppm | ^74^ |
| a/c-Ru/Ti-RuO_2_ | 26% | 2000 | 1000 ppm | ^75^ |
| HEA SNWs | 26.4 | 2000 | 1000 ppm | ^76^ |
| Rh_2_Sb | 45.5% | 2000 | 1000 ppm | ^77^ |
| PtRu_3_/C | 31.4 | 3500 | 1000 ppm | ^78^ |
| i-ZnIn-PR/C | 15.1% | 5000 | 1000 ppm | ^37^ |
| O-Pt_3_In/rGO | 19.93% | 1800 | 2000 ppm | ^79^ |
| Ru/RuO_2_/C in-plane heterostructures | 23.5% | 300 | 10000 ppm | ^49^ |

**D. Supplementary References**

[1] Y. Zhang, C. Ma, X. Zhu, K. Qu, P. Shi, L. Song, J. Wang, Q. Lu, A. Wang, Hetero-interface manipulation in MoOx@Ru to evoke industrial hydrogen production performance with current density of 4000 mA cm^−2^, Adv. Energy Mater. 13 (2023) 2301492.

[2] X. Wu, Z. Wang, D. Zhang, Y. Qin, M. Wang, Y. Han, T. Zhan, B. Yang, S. Li, J. Lai, L. Wang, Solvent-free microwave synthesis of ultra-small Ru-Mo_2_C@CNT with strong metal-support interaction for industrial hydrogen evolution, Nat. Commun. 12 (2021) 4018.

[3] X. Mu, X. Gu, S. Dai, J. Chen, Y. Cui, Q. Chen, M. Yu, C. Chen, S. Liu, S. Mu, Breaking the symmetry of single-atom catalysts enables an extremely low energy barrier and high stability for large-current-density water splitting, Energy Environ. Sci. 15 (2022) 4048-4057.

[4] Z. Xu, J. Zhu, Z. Shu, Y. Xia, R. Chen, S. Chen, Y. Wang, L. Zeng, J. Wang, Y. Cai, S. Chen, F. Huang, H. Wang, Phosphorus-induced anti-growth of ruthenium clusters-single atoms for ultra-stable hydrogen evolution over 100,000 cycles, Joule 8 (2024) 2542-4351.

[5] L. Wu, F. Zhang, S. Song, M. Ning, Q. Zhu, J. Zhou, G. Gao, Z. Chen, Q. Zhou, X. Xing, T. Tong, Y. Yao, J. Bao, L. Yu, Luo, S. Chen, Z. Ren, Efficient alkaline water/seawater hydrogen evolution by a nanorod‐nanoparticle‐structured Ni‐MoN catalyst with fast water‐dissociation kinetics, Adv. Mater. 34 (2022) 2201774.

[6] X. Xiao, X. Wang, X. Jiang, S Song, D. Huang, L. Yu, Y. Zhang, S. Chen, M. Wang, Y. Shen, Z. Ren, In situ growth of Ru nanoparticles on (Fe, Ni)(OH)_2_ to boost hydrogen evolution activity at high current density in alkaline media, Small Methods 4 (2020) 1900796.

[7] W. Yu, H. Huang, Y. Qin, D. Zhang, Y. Zhang, K. Liu, Y. Zhang, J. Lai, L. Wang, The synergistic effect of pyrrolic‐N and pyridinic‐N with Pt under strong metal‐support interaction to achieve high‐performance alkaline hydrogen evolution, Adv. Energy Mater. 12 (2022) 2200110.

[8] X. Wang, G. Long, B. Liu, Z. Li, W. Gao, P. Zhang, H. Zhang, X. Zhou, R. Duan, W. Hu, C. Li, Rationally modulating the functions of Ni_3_Sn_2_‐NiSnO_x_ nanocomposite electrocatalysts towards enhanced hydrogen evolution reaction, Angew. Chem. Int. Ed. 62 (2023) e202301562.

[9] Y. Wang, G. Qian, Q. Xu, H. Zhang, F. Shen, L. Luo, S. Yin, Industrially promising IrNi-FeNi_3_ hybrid nanosheets for overall water splitting catalysis at large current density. Appl. Catal. B-Environ. 286 (2021) 119881.

[10] S. Parvin, A. Kumar, A. Ghosh, S. Bhattacharyya, An earth-abundant bimetallic catalyst coated metallic nanowire grown electrode with platinum-like pH-universal hydrogen evolution activity at high current density, Chem. Sci. 11 (2020) 3893-3902.

[11] P. Zhai, Y. Zhang, Y. Wu, J. Gao, B. Zhang, S. Cao, Y. Zhang, Z. Li, L. Sun, J. Hou, Engineering active sites on hierarchical transition bimetal oxides/sulfides heterostructure array enabling robust overall water splitting, Nat. Commun. 11 (2020) 5462.

[12] P. Fang, M. Zhu, J. Liu, Z. Zhu, J. Hu, X. Xu, Making ternary‐metal hydroxysulfide catalyst via cathodic reconstruction with ion regulation for industrial‐level hydrogen generation, Adv. Energy Mater. 13 (2023) 2301222.

[13] Q. Hu, K. Gao, X. Wang, H. Zheng, J. Cao, L. Mi, Q. Huo, H. Yang, J. Liu, C. He, Subnanometric Ru clusters with upshifted D band center improve performance for alkaline hydrogen evolution reaction, Nat. Commun. 13 (2022) 3958.

[14] L. Wang, Y. Hao, L. Deng, F. Hu, S. Zhao, L. Li, S. Peng, Rapid complete reconfiguration induced actual active species for industrial hydrogen evolution reaction, Nat. Commun. 13 (2022) 5785.

[15] H. Fu, M. Zhou, P. Liu, P Liu, H Yin, K. Sun, H. Yang, M. AlMamun, P. Hu, H. Wang, H. Zhao, Hydrogen spillover-bridged Volmer/Tafel processes enabling ampere-level current density alkaline hydrogen evolution reaction under low overpotential, J. Am. Chem. Soc. 144 (2022) 6028-6039.

[16] T. Luo, J. Huang, Y. Hu, C. Yuan, J. Chen, L. Cao, K. Kajiyoshi, Y. Liu, Y. Zhao, Z. Li, Y. Feng, Fullerene lattice‐confined Ru nanoparticles and single atoms synergistically boost electrocatalytic hydrogen evolution reaction, Adv. Funct. Mater. 33 (2023) 2213058.

[17] Y. Zhang, K. E. Arpino, Q. Yang, N. Kikugawa, D. A. Sokolov, C. W. Hicks, J. Liu, C. Felser, G. Li, Observation of a robust and active catalyst for hydrogen evolution under high current densities, Nat. Commun. 13 (2022) 7784.

[18] C. Yang, W. Zhong, K. Shen, Q. Zhang, R. Zhao, H. Xiang, J. Wu, X. Li, N. Yang, Electrochemically reconstructed Cu‐FeOOH/Fe_3_O_4_ catalyst for efficient hydrogen evolution in alkaline media, Adv. Energy Mater. 12 (2022) 2200077.

[19] G. Zhang, A. Wang, L. Niu, W. Gao, W. Hu, Z. Liu, R. Wang, J. Chen, Interfacial engineering to construct antioxidative Pd_4_S/Pd_3_P_0.95_ heterostructure for robust hydrogen production at high current density, Adva. Energy Mater. 12 (2022) 2103511.

[20] C. Liu, G. Pan, N. Liang, S .Hong, J. Ma, Y. Liu, Ir single atom catalyst loaded on amorphous carbon materials with high HER activity, Adv. Sci. 9 (2022) 2105392.

[21] P. Zhai, M. Xia, Y. Wu, G. Zhang, J. Gao, B. Zhang, S. Cao, Y. Zhang, Z. Li, Z. Fan, C. Wang, Engineering single-atomic ruthenium catalytic sites on defective nickel-iron layered double hydroxide for overall water splitting, Nat. Commun. 12 (2021) 4587.

[22] T. Chao, W. Xie, Y. Hu, G. Yu, T. Zhao, C. Chen, Z. Zhang, X. Hong, H. Jin, D. Wang, W. Chen, X. Li, Reversible hydrogen spillover at the atomic interface for efficient alkaline hydrogen evolution, Energy Environ. Sci. 17 (2024) 1397-1406.

[23] L. Zeng, Z. Zhao, Q. Huang, C. Zhou, W. Chen, K. Wang, M. Li, F. Lin, H. Luo, Y. Gu, L. Li, S. Zhang, Single-atom Cr-N_4_ sites with high oxophilicity interfaced with Pt atomic clusters for practical alkaline hydrogen evolution catalysis, J. Am. Chem. Soc. 145 (2023) 21432-21441.

[24] J. Yu, Y. Dai, X. Wu, Z. Zhang, Q. He, C. Cheng, Z. Wu, Z. Shao, M. Ni, Ultrafine ruthenium-iridium alloy nanoparticles well-dispersed on N-rich carbon frameworks as efficient hydrogen-generation electrocatalysts, Chem. Eng. J. 417 (2021) 128105.

[25] Y. Zhao,P. V. Kumar, X. Tan, X. Lu, X. Zhu, J. Jiang, J. Pan, S. Xi, H. Y. Yang, Z. Ma, T. Wan, D. Chu, Modulating Pt-O-Pt atomic clusters with isolated cobalt atoms for enhanced hydrogen evolution catalysis, Nat. Commun. 13 (2022) 2430.

[26] Z. Zhang, G. Liu, X. Cui, B. Chen, Y. Zhu, Y. Gong, F. Saleem, S. Xi, Y. Du, A. Borgna, Z. Lai, Q. Zhang, B. Li, Y. Zong, Y. Han, L. Gu, H. Zhang, Crystal phase and architecture engineering of lotus‐thalamus‐shaped Pt‐Ni anisotropic superstructures for highly efficient electrochemical hydrogen evolution, Adv. Mater. 30 (2018) 1801741.

[27] Z. Xing, D. Wang, T. Meng, X. Yang, Superb hydrogen evolution by a Pt nanoparticle-decorated Ni_3_S_2_ microrod array, ACS appl. Mater. 12 (2020) 39163-39169.

[28] Q. He, Y. Zhou, H. Shou, X. Wang, P. Zhang, W. Xu, S. Qiao, C. Wu, H. Liu, D. Liu, S. Chen, R. Long, Synergic reaction kinetics over adjacent ruthenium sites for superb hydrogen generation in alkaline media, Adv. Mater. 34 (2022) 2110604.

[29] A. Nairan, C. Liang, S. W. Chiang, Y. Wu, P. Zou, U. Khan, W. Liu, F. Kang, S. Guo, J. Wu, C. Yang, Proton selective adsorption on Pt-Ni nano-thorn array electrodes for superior hydrogen evolution activity, Energy Environ. Sci. 14 (2021) 1594-1601.

[30] H. Huang, H. Jung, S. Li, S. Kim, J. W. Han, J. Lee, Activation of inert copper for significantly enhanced hydrogen evolution behaviors by trace ruthenium doping, Nano Energy 92 (2022) 106763.

[31] T. Chao, W Xie, Y. Hu, G. Yu, T. Zhao, C. Chen, Z. Zhang, X. Hong, H. Jin, D. Wang, W. Chen, X. Li, Reversible hydrogen spillover at the atomic interface for efficient alkaline hydrogen evolution, Energy Environ. Sci. 17 (2024) 1397-1406.

[32] Y. R. Hong, S. Dutta, S. W. Jang, O. Okello, H. Im, S. Y. Choi, J. W. Han, I. S. Lee, Crystal facet-manipulated 2D Pt nanodendrites to achieve an intimate heterointerface for hydrogen evolution reactions, J. Am. Chem. Soc. 144 (2022) 9033-9043.

[33] P. Kuang, Y. Wang, B. Zhu, F. Xia, C. W. Tung, J. Wu, H. M. Chen, J. Yu, Pt single atoms supported on N‐doped mesoporous hollow carbon spheres with enhanced electrocatalytic H_2_‐evolution activity, Adv. Mater. 33 (2021) 2008599.

[34] X. K. Wan, H. B. Wu, B. Y. Guan, D. Luan, X. W. Lou, Confining sub‐nanometer Pt clusters in hollow mesoporous carbon spheres for boosting hydrogen evolution activity, Adv. Mater. 32 (2020) 1901349.

[35] B. Zhang, C. Zhu, Z. Wu, E. Stavitski, Y. H. Lui, T. H. Kim, H. Liu, L. Huang, X. Luan, L. Zhou, K. Jiang, Integrating Rh species with NiFe-layered double hydroxide for overall water splitting, Nano Lett. 20 (2019) 136-144.

[36] J. Dai, Y. Zhu, H. A. Tahini, Q. Lin, Y. Chen, D. Guan, C. Zhou, Z. Hu, H. J. Lin, T. S. Chan, C. T. Chen, Single-phase perovskite oxide with super-exchange induced atomic-scale synergistic active centers enables ultrafast hydrogen evolution, Nat. Commun. 11(2020) 5657.

[37] Z. Huang, S. Hu, M. Sun, Y. Xu, S. Liu, R. Ren, L. Zhuang, T. Chan, Z. Hu, T. Ding, J. Zhou, L. Liu, M. Wang, Y. Huang, N. Tian, B. Huang, H. Huang, Implanting oxophilic metal in PtRu nanowires for hydrogen oxidation catalysis, Nat. Commun. 15 (2024) 1097.

[38] H. Liu, J. Fu, H. Li, J. Sun, X. Liu, Y. Qiu, X. Peng, Y. Liu, H. Bao, L. Zhuo, R. Cao, S. Zhang, J. Luo, Single palladium site in ordered porous heteroatom-doped carbon for high-performance alkaline hydrogen oxidation, Appl. Catal. B-Environ. 306 (2022) 121029.

[39] Y. Zhao, X. Wang, Z. Li, P. Zhao, C. Tao, G. Chen, W. Luo, Enhanced catalytic activity of Ru through N modification toward alkaline hydrogen electrocatalysis, Chin. Chem. Lett. 33 (2022) 1065-1069.

[40] T. Zhao, D. Xiao, Y. Chen, X. Tang, M. Gong, S. Deng, X. Liu, J. Ma, X. Zhao, D. Wang, Boosting alkaline hydrogen electrooxidation on an unconventional fcc-Ru polycrystal, J. Energy Chem. 61 (2021) 15-22.

[41] Y.Cong, L. Chen, M. Liu, H. Wang, L. Zhang, Q. Zhao, C. Li, Synergistic coupling of defective Ru nanoclusters with oxygen ligand steered Ru single atoms modifying interfacial water structure and key intermediates bonding toward efficient hydrogen energy conversion, Chem. Eng. J. 495 (2024) 153433.

[42] L. Li, C. Liu, S. Liu, J. Wang, J. Han, T. S. Chan, Y. Li, Z. Hu, Q. Shao, Q. Zhang, X. Huang, Phase engineering of a ruthenium nanostructure toward high-performance bifunctional hydrogen catalysis, ACS Nano 16 (2022) 14885-14894.

[43] J. Jiang, S. Tao, Q. He, J. Wang, Y. Zhou, Z. Xie, W. Ding, Z. Wei, Interphase-oxidized ruthenium metal with half-filled d-orbitals for hydrogen oxidation in an alkaline solution, J. Mater. Chem. A 8 (2020) 10168-10174.

[44] H. Shi, Y. Yang, P. Meng, J. Yang, W. Zheng, P. Wang, Y. Zhang, X. Chen, Z. Cheng, C. Zong, D. Wang, Q. Chen, Local charge transfer unveils antideactivation of Ru at high potentials for the alkaline hydrogen oxidation reaction, J. Am. Chem. Soc. 146 (2024) 16619-16629.

[45] B. Zhang, G. Zhao, J. Wang, D. Liu, Y. Chen, L. Xia, M. Gao, Y. Liu, W. Sun, H. Pan, Atomically dispersed chromium coordinated with hydroxyl clusters enabling efficient hydrogen oxidation on ruthenium, Nat. Commun. 13 (2022) 5894.

[46] L. Wang, Z. Xu, C.H. Kuo, J. Peng, F. Hu, L. Li, H.Y. Chen, J. Wang, S. Peng, Stabilizing low‐valence single atoms by constructing metalloid tungsten carbide supports for efficient hydrogen oxidation and evolution, Angew. Chem. Int. Ed. 62 (2023) e202311937.

[47] L. Jing, G. Jie, W. Yu, H. Ren, X. Cui, X. Chen, L. Jiang, A unique sandwich-structured Ru-TiO/TiO_2_@NC as an efficient bi-functional catalyst for hydrogen oxidation and hydrogen evolution reactions, Chem. Eng. J. 472 (2023) 145009.

[48] J. Zhang, G. Ren, D. Li, Q. Kong, Z. Hu, Y. Xu, S. Wang, Interface engineering of snow-like Ru/RuO_2_ nanosheets for boosting hydrogen electrocatalysis, Sci. Bull. 67 (2022) 2103-2111.

[49] X. Zhang, L. Xia, G. Zhao, B. Zhang, Y. Chen, J. Chen, M. Gao, Y. Jiang, Y. Liu, H. Pan, W. Sun, Fast and durable alkaline hydrogen oxidation reaction at the electron‐deficient ruthenium-ruthenium oxide interface, Adv. Mater. 35 (2023) 2208821.

[50] Y. Yang, Y. Huang, S. Zhou, Y. Liu, L. Shi, T. T. Isimjan, X. L. Yang, Delicate surface vacancies engineering of Ru doped MOF-derived Ni-NiO@C hollow microsphere superstructure to achieve outstanding hydrogen oxidation performance, J. Energy Chem. 72 (2022) 395-404.

[51] Y. Zhao, D. Wu, W. Luo, Correlating alkaline hydrogen electrocatalysis and hydroxide binding energies on Mo-modified Ru catalysts, ACS Sustain. Chem. Eng. 10 (2022) 1616-1623.

[52] L. Su, Y. Jin, D. Gong, X. Ge, W. Zhang, X. Fan, W. Luo, The role of discrepant reactive intermediates on Ru‐Ru_2_P heterostructure for pH‐universal hydrogen oxidation reaction, Angew. Chem. Int. Ed. 62 (2023) e202215585.

[53] Y. Zhao, F. Yang, W. Zhang, Q. Li, X. Wang, L. Su, X. Hu, Y. Wang, Z. Wang, L. Zhuang, S. Chen, W. Luo, High-performance Ru_2_P anodic catalyst for alkaline polymer electrolyte fuel cells, CCS Chem. 4 (2022) 1732-1744.

[54] C. P. Huang, M.C. Tsai, X. M. Wang, H. S. Cheng, Y. H. Mao, C. J. Pan, J. Lin, W. N. Su, Engineering heterometallic bonding in bimetallic electrocatalysts: towards optimized hydrogen oxidation and evolution reactions, Catal. Sci. Technol. 10 (2020) 893-903.

[55] Y. Li, C. Yang, C. Ge, N. Yao, J. Yin, W. Jiang, H. Cong, G. Cheng, W. Luo, L. Zhuang, Electronic modulation of Ru nanosheet by d-d orbital coupling for enhanced hydrogen oxidation reaction in alkaline electrolytes, Small 18 (2022) 2202404.

[56] Y. Dong, Q. Sun, C. Zhan, J. Zhang, H. Yang, T. Cheng, Y. Xu, Z. Hu, C. W. Pao, H. Geng, X. Huang, Lattice and surface engineering of ruthenium nanostructures for enhanced hydrogen oxidation catalysis, Adv. Funct. Mater. 33 (2023) 2210328.

[57] Y. Dong, Z. Zhang, W. Yan, X. Hu, C. Zhan, Y. Xu, X. Huang, Pb‐Modified ultrathin RuCu nanoflowers for active, stable, and CO‐resistant alkaline electrocatalytic hydrogen oxidation, Angew. Chem. Int. Ed. 62 (2023) e202311722.

[58] Z. Yang, W. Lai, B. He, J. Wang, F. Yu, Q. Liu, M. Liu, S. Zhang, W. Ding, Z. Lin, H. Huang, Tailoring interfacial chemistry of defective carbon‐supported Ru catalyst toward efficient and CO‐tolerant alkaline hydrogen oxidation reaction, Adv. Energy Mater. 13 (2023) 2300881.

[59] Y. Liu, L. Cheng, Y. Huang, Y. Yang, X. Rao, S. Zhou, T. T. Isimjan, X. Yang, Electronic modulation and mechanistic study of Ru‐decorated porous Cu‐rich cuprous oxide for robust alkaline hydrogen oxidation and evolution reactions, ChemSusChem 16 (2023) e202202113.

[60] C. Yang, Y. Li, J. Yue, H. Cong, W. Luo, Promoting water formation in sulphate-functionalized Ru for efficient hydrogen oxidation reaction under alkaline electrolytes, Chem. Sci. 14 (2023) 6289-6294.

[61] J. Wang, J. Liu, B. Zhang, J. Gao, G. Liu, X. Cui, J. X. Liu, L. Jiang, Amine-ligand modulated ruthenium nanoclusters as a superior bi-functional hydrogen electrocatalyst in alkaline media, J. Mater. Chem. A 9 (2021) 22934-22942.

[62] Y. Yang, X. Shao, S. Zhou, P. Yan, T. T. Isimjan, X. Yang, Interfacial electronic coupling of NC@WO_3_‐W_2_C decorated Ru clusters as a reversible catalyst toward electrocatalytic hydrogen oxidation and evolution reactions, ChemSusChem 14 (2021) 2992-3000.

[63] G. Meng, H. Cao, T. Wei, Q. Liu, J. Fu, S. Zhang, J. Luo, X. Liu, Highly dispersed Ru clusters toward an efficient and durable hydrogen oxidation reaction, Chem. Commun. 58 (2022) 11839-11842.

[64] L. Su, X. Fan, Y. Jin, H. Cong, W. Luo, Hydroxyl‐binding energy‐induced kinetic gap narrowing between acidic and alkaline hydrogen oxidation reaction on intermetallic Ru_3_Sn_7_ catalyst, Small 19 (2023) 2207603.

[65] H. Liu, Z. Zhang, M. Li, Y. Li, Y. Kuang, X. Sun, Ru-doped WO_3_ enabling efficient hydrogen oxidation reaction in alkaline media, Nanoscale 15 (2023) 12064-12070.

[66] Q. Wu, W. Yang, X. Wang, W. Zhu, S. Lv, Y. Zhou, T. Chen, S. Liu, W. Li, Z. Chen, Inherent vacancy of compressive Ru nanoparticles accelerate electro-catalytic hydrogen energy conversion, Appl. Catal. B-Environ. 335 (2023) 122896.

[67] Y. Xie, B. Lian, S. Deng, Q. Lin, K. Wang, Y. Zheng, Z. Zhuang, Y. Liu, K. Sun, W. Yan, J. Zhang, Advanced Ru/Ti_4_O_7_ catalyst for tolerating CO and H_2_S poisoning to hydrogen oxidation reaction, Int. J. Hydrogen Energy 65 (2024) 205-214.

[68] J. Liu, B. Zhang, Y. Fo, J. Gao, W. Yu, H. Ren, X. Cui, X. Zhou, L. Jiang, Unique Ru nanoclusters confined in carbon molecular sieve coatings with tailoring sub-4Å ultramicropores as a highly efficient and CO-tolerant hydrogen oxidation electrocatalyst, Chem. Eng. J. 468 (2023) 143438.

[69] Y. Men, D. Wu, Y. Hu, L. Li, P. Li, S. Jia, J Wang, G. Cheng, S. Chen, W. Luo, Understanding alkaline hydrogen oxidation reaction on PdNiRuIrRh high‐entropy‐alloy by machine learning potential, Angew. Chem. Int. Ed. 62 (2023) e202217976.

[70] L. Han, P. Ou, W. Liu, X. Wang, H. T. Wang, R. Zhang, C. W. Pao, X. Liu, W. F. Pong, J. Song, Design of Ru-Ni diatomic sites for efficient alkaline hydrogen oxidation, Sci. Adv. 8 (2022) eabm3779.

[71] L. Wei, W. Yan, Z. Huang, R. Li, Q. Kong, W. H. Huang, C. W. Pao, Z. Hu, H. Lin, N. Chen, Y. Xu, H. Geng, X. Q. Huang, Phase and interface engineering of a Ru-Sn nanocatalyst for enhanced alkaline hydrogen oxidation reaction, Energy Environ. Sci. 17 (2024) 5922-5930.

[72] Y. Zhou, Z. Xie, J. Jiang, J. Wang, X. Song, Q. He, W. Ding, Z. Wei, Lattice-confined Ru clusters with high CO tolerance and activity for the hydrogen oxidation reaction, Nat. Catal. 3 (2020) 454-462.

[73] X. Mu, X. Zhang, Z. Chen, Y. Gao, M. Yu, D. Chen, H. Pan, S. Liu, D. Wang, S. Mu, Constructing symmetry-mismatched Ru_x_Fe_3-x_O_4_ heterointerface-supported Ru clusters for efficient hydrogen evolution and oxidation reactions, Nano Lett. 24 (2024) 1015-1023.

[74] X. Wang, L. Zhao, X. Li, Y. Liu, Y. Wang, Q. Yao, J. Xie, Q. Xue, Z. Yan, X. Yuan, W. Xing, Atomic-precision Pt_6_ nanoclusters for enhanced hydrogen electro-oxidation, Nat. Commun. 13 (2022) 1596.

[75] L. Wei, N. Fang, F. Xue, S. Liu, W. H. Huang, C. W. Pao, Z. Hu, Y. Xu, H. Geng, X. Huang, Amorphous-crystalline RuTi nanosheets enhancing OH species adsorption for efficient hydrogen oxidation catalysis, Chem. Sci. 15 (2024) 3928-3935.

[76] C. Zhan, Y. Xu, L. Bu, H. Zhu, Y. Feng, T. Yang, Y. Zhang, Z. Yang, B. Huang, Q. Shao, X. Huang, Subnanometer high-entropy alloy nanowires enable remarkable hydrogen oxidation catalysis, Nat. Commun. 12 (2021) 6261.

[77] Y. Zhang, G. Li, Z. Zhao, L. Han, Y. Feng, S. Liu, B. Xu, H. Liao, G. Lu, H. L. Xin, X. Huang, Atomically isolated Rh sites within highly branched Rh_2_Sb nanostructures enhance bifunctional hydrogen electrocatalysis, Adv. Mater. 33 (2021) 2105049.

[78] J. Zhang, X. Qu, L. Shen, G. Li, T. Zhang, J. Zheng, L. Ji, W. Yan, Y. Han, X. Cheng, Y. Jiang, S. Sun, Engineering the near‐surface of PtRu_3_ nanoparticles to improve hydrogen oxidation activity in alkaline electrolyte, Small 17 (2021) 2006698.

[79] J. Wu, X. Gao, G. Liu, X. Qiu, Q. Xia, X. Wang, W. Zhu, T. He, Y. Zhou, K. Feng, J. Wang, H. Huang, Y. Liu, M. Shao, Z. Kang, Immobilizing ordered oxophilic indium sites on platinum enabling efficient hydrogen oxidation in alkaline electrolyte, J. Am. Chem. Soc. 146 (2024) 20323-20332.
